# Comparison of the efficacy and safety of third-line treatments for advanced gastric cancer: A systematic review and network meta-analysis

**DOI:** 10.3389/fonc.2023.1118820

**Published:** 2023-03-01

**Authors:** Chan Zhang, Yaoxian Xiang, Jing Wang, Dong Yan

**Affiliations:** Department of Oncology, Beijing Luhe Hospital Affiliated to Capital Medical University, Beijing, China

**Keywords:** gastric cancer (GC), gastroesophageal junction carcinoma (GEJC), third-line treatment, nivolumab, apatinib, ADC, chemotherapy, network meta-analysis (NMA)

## Abstract

**Background:**

Many options for third-line treatment of advanced gastric cancer (GC) or gastroesophageal junction carcinoma (GEJC) have been developed. Therapies including immunotherapy (nivolumab), chemotherapy (irinotecan, FTD/TPI), targeted therapy (apatinib), and antibody drug conjugates (ADC) have shown to increase the survival rates in patients, but few studies have compared the relative efficacy of these treatments. Here, we compared the efficacies of these regimens using network meta-analysis (NMA) to provide guides in selecting the best regimen and formulating a precise individualized treatment plan.

**Methods:**

The published RCTs of phase II/III in PubMed, the Cochrane Central Register of Controlled Trials, and Embase were searched. The median overall survival (mOS) was the primary outcome of NMA, and the other outcomes were median progression-free survival (mPFS), disease control rate (DCR) (proportion of patients with confirmed CR, PR, or stable disease (SD)) and incidence of grade 3 or above adverse events (≥3AEs).

**Results:**

Five phase II/III RCTs involving 1674 patients and 7 treatment regimens were analyzed. It showed that Trastuzumab Deruxtecan (DS-8201) prolonged the OS of patients significantly comparing with chemotherapy (HR: 0.59; 95% CI: 0.39-0.89) for the overall population. DS-8201 (HR: 0.27; 95% CI: 0.17-0.42) and chemotherapy (HR: 0.57; 95% CI: 0.47-0.7) improved the PFS significantly over nivolumab. Apatinib (RR: 3.04; 95% CI: 1.65-5.95) and DS-8201 (RR: 2.67; 95% CI: 1.51-4.83) were more effective than nivolumab in improving DCR. DS-8201 achieved greater OS benefits compared to chemotherapy (HR: 0.59; 95% CI: 0.39-0.88) for patients who were HER2-positive. We ranked the Bayesian surface under the cumulative ranking curve according to OS benefit, and showed that ADC ranked first for the general patient population and for patients with a HER2-positive diagnosis, intestinal histopathology, previous gastrectomy history, gastric origination cancer, ages over 65 and ECOG PS=0/1, followed by nivolumab and apatinib. For patients with GEJC, nivolumab ranked first.

**Conclusions:**

Nivolumab, apatinib, chemotherapy, and ADC all improved the OS of GC/GEJC patients significantly. ADC may be the best option for the overall population of GC, as well as for patients with HER2-overexpression, intestinal histopathology, previous gastrectomy history, gastric origination cancer, ages over 65 and ECOG PS=0/1, followed by nivolumab and apatinib. Nivolumab may be the first treatment option for GEJC patients.

**Systematic review registration:**

https://www.crd.york.ac.uk/prospero, identifier CRD42022364714.

## Introduction

GC and GEJC have become one of the most frequently diagnosed malignant tumors in recent years, and they ranked the fourth in tumor-caused death (1), with an incidence of 29.3/100,000 and a mortality rate of 21.2/100,000 in China (2). Despite the significant progress in the options for effective surgical and systemic treatments, the overall 5-year survival rate of GC remains at below 30%, and the median OS of advanced gastric cancer (AGC) is only 9-10 months (3, 4). The development of more effective multidisciplinary evaluation and treatments for GC/GEJC is needed.

At present, first-line standard treatments recommended by National Comprehensive Cancer Network (NCCN) guidelines are fluorouracil-based options, combined with standard chemotherapy using platinum and/or taxane, and with or without anti-HER2 drugs depending on HER2 expression status (5). In addition, depending on the expression status of PD-L1, immunotherapy options may be added as well. Second-line treatment is mostly recommended as monotherapy. However, third-line treatment includes many different options after second-line treatment fails. At present, options for third-line treatment of GC or GEJC include targeted therapy (apatinib), immunotherapy (nivolumab), and chemotherapy (irinotecan, FTD/TPI) (6, 7). Despite the survival benefits of all these regimens for GC patients, the objective response rate (ORR) of tumors remains low (2.84%-11.6%) (8–11). Surprisingly, some new ADC drugs have shown great efficacy and safety. For example, trastuzumab deruxtecan (DS-8201) has emerged in recent years as an effective treatment for HER2-positive GC patients. Although DS-8201 may cause interstitial lung disease in some patients with an incidence rate of approximately 10%, its safety profile remains manageable (12). The new ADC drug RC48 is produced by coupling recombinant human anti-HER2 antibody with monomethyl auristatin E (a microtubule inhibitor) through a cleavable linker. In some RCTs, RC48 also showed great anti-solid tumor activity against GC. In addition, it has shown high efficacy in patients with low expression of HER2 (IHC 2+/FISH-) and HER2 overexpression (IHC 2+/FISH+ or HER2 IHC 3+) (13, 14). 2.5 mg/kg RC48 was administered every two weeks with a single intravenous infusion to treat patients with HER2 overexpression in a phase II single-arm RCT. The treated participants showed a median OS of 7.9 months (95% CI: 6.7-9.9) and a median PFS of 4.1 months (95% CI: 3.7-4.9) (15).

The overall prognosis of advanced GC is relatively poor. Clinical research on traditional chemotherapeutic agents has not identified effective drugs, the choice of targeted drugs is limited, and the efficacy of immunotherapy alone is insufficient. Therefore, we analyzed several third-line treatment options by comparing their efficacy and safety, to provide a guide in choosing the best third-line treatment for GC.

## Materials and methods

### Literature search strategies

This NMA was performed according to the PRISMA extension statement (**Supplementary Table 1**). Publication on the third-line treatments for advanced GC/GEJC in PubMed, Embase, and Cochrane Library and Medline ISI (January 1, 2005 to November 31, 2021) were searched using the search strategy shown in the **Supplemental Table 2**. We also reviewed abstracts of major conferences (2018-2022) including the European Society of Medical Oncology (EMSO), American Society of Clinical Oncology (ASCO), Chinese Society of Clinical Oncology Collaborative Committee (CSCO), and American Association for Cancer Research (AACR).

### Inclusion criteria

We selected published English-language reports of RCTs of phase II/III that compared at least two third-line treatment regimens. The patients who were included in the study were required to have advanced (stage IV) GC/GEJC diagnosed histologically. In addition, the hazard ratio (HR) and 95% confidence interval (CI) with OS and PFS were available.

### Exclusion criteria

We excluded phase I clinical trials and those with incomplete data reports. Studies that tested only adjuvant therapy, maintenance therapy, or first-line and second-line therapy were also excluded. We also excluded articles related to tumor vaccine treatment.

### Data extraction and risk of bias assessment

We first extracted relevant information of included studies, such as study title, publication year, first author, number of study subjects and baseline characteristics, and indicators of OS, PFS, ORR, DCR as well as ≥3AEs. The risk of bias in RCTs was subsequently determined using the Cochrane Risk of Bias Tool, which included the randomization process, missing outcome data, measurement of outcomes, deviation from the intended intervention, and selection of reported outcomes. RCTs were rated as low, high, or some concern of bias based on this evaluation criteria. For non-RCT, we used the Newcastle-Ottawa scale for quality assessment, which include the exposed cohort, non-exposed cohort, ascertainment of exposure, outcome of interest, comparability, assessment of outcome, length of follow-up, adequacy of follow up. A total score of 5 or more is considered high quality study (16). Extraction of the data and assessment of the risk of bias were carried out by two independent investigators (XYX and ZC).

### Statistical analysis

Q-test and I^2^ statistics were used to assess the heterogeneity among studies. Heterogeneity among studies could be considered statistically significant if I^2^ ≥ 50% or P < 0.05. If I^2^ values were less than 50%, studies could be considered having low to moderate heterogeneity and a random effect model could be applied for statistical analysis (17). For the HR, relative risk (RR), and corresponding 95% CI of the outcome indicators including OS, PFS, DCR, and ≥3AEs, we applied fixed and random models separately to pool and then compare them by the deviance information criterion (DIC). We then chose the fixed model when the difference in DIC between the random and fixed models was less than 5 (18). Bayesian NMA was carried out using the JAGS and GEMTC packages in R.4.2.0 and Markov chain Monte Carlo simulation technology (19). Each analysis involved 150,000 sample iterations with 100,000 burn-in cycles and a thinning interval of 10. In addition, we used tracking maps and Brooks-Gelman-Rubin diagnostics for visual inspection to help determine the model convergence (20). The network diagrams produced with Stata 16.0 showed the comparative relationships between the various treatments more directly. We calculated the surface under the cumulative ranking (SUCRA) curve to estimate the probability that each treatment method was at each rank. A higher SUCRA value represented a greater possibility that a treatment would be treated as the top choice (21).

## Results

### Network meta-analysis study characteristics

**Figure 1:**
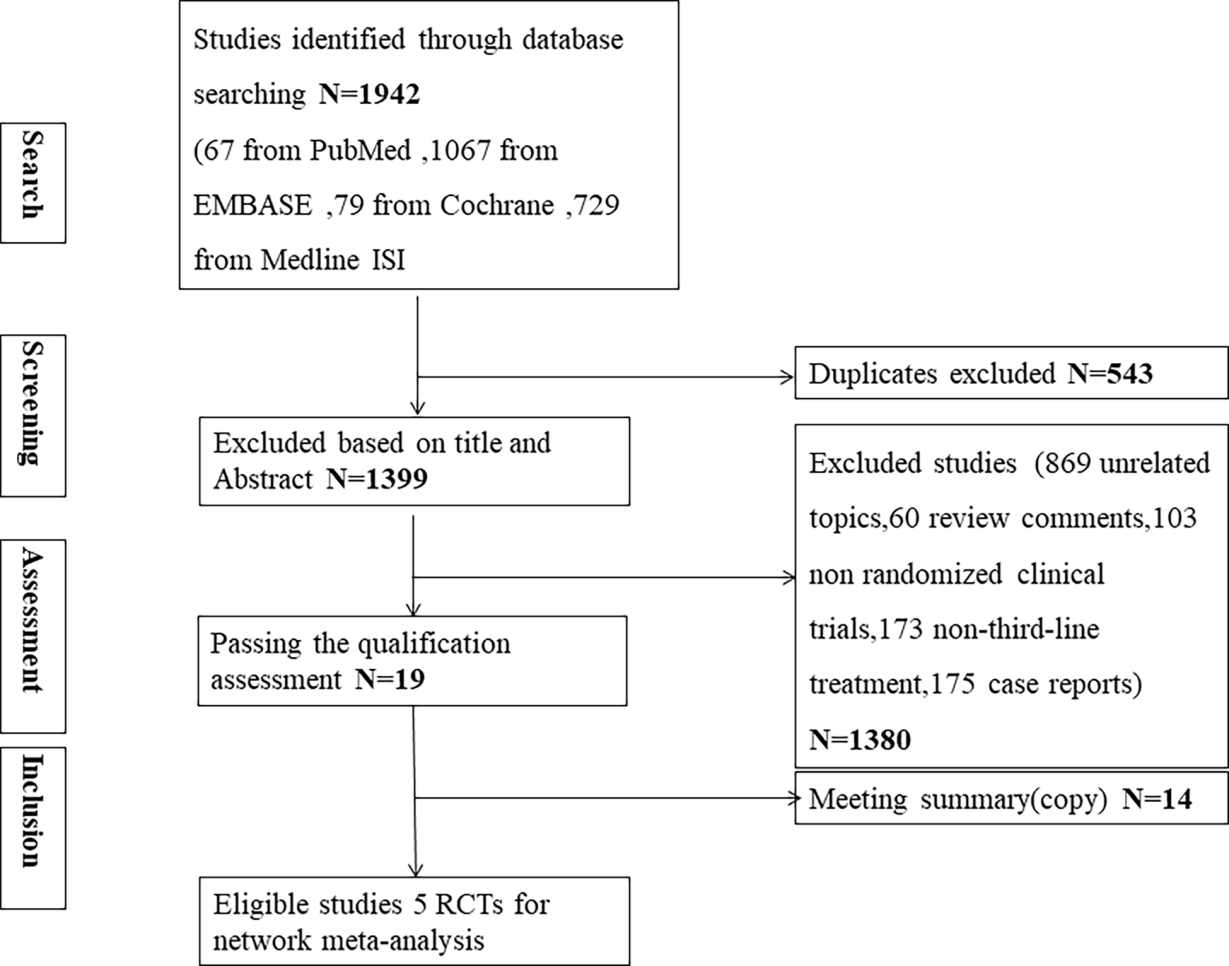
Study selection.

After the screening process as shown in **Figure 1**, five phase II/III eligible RCTs (10–12, 22, 23) in our review with a total of 1674 patients and 7 different treatments were included. The treatments included immunotherapy (nivolumab), chemotherapy (trifluridine/tipiracil, irinotecan, paclitaxel), targeted therapy (apatinib), and ADC (trastuzumab deruxtecan, (DS-8201) and virtuximab (RC48-ADC)). **Table 1** shows the basic characteristics of included RCTs. Our NMA satisfied the assumption of transitivity that the population baseline was stable across studies with different interventions. (**Supplementary Figure 9**)

**Table 1:** Baseline characteristics of studies included in the systematic review with Bayesian network meta-analysis of third-line treatments for advanced gastric cancer.

Study (year)	Phase	Study Design	Sample size	Median age	Male/Female	Intervention arm	Control arm	Tumor type	Reported outcomes
ATTRACTION-2 (22)	III	RCT	330/163	62/61	348/145	Intravenous infusion of nivolumab every 2 weeks for 6 weeks (one treatment cycle) (3 mg/kg)	Intravenous infusion of placebo every 2 weeks for 6 weeks (one treatment cycle) (3 mg/kg)	GC/GEJC	OS,PFS,ORR,DCR,AE
Jin Li (10)	III	RCT	176/91	58/58	201/66	Oral apatinib 850 mg in tablet form once daily	Oral apatinib 850 mg in apatinib matching placebo once daily	GC/GEJC	OS,PFS,ORR,DCR,AE
Jin Li (23)	II	RCT	47/48	55/65	75/20	Oral apatinib 850 mg once daily	Oral placebo 850 mg once daily	GC/GEJC	OS,PFS,ORR,DCR,AE
DESTINY-Gastric01 (12)	II	RCT	125/62	65/66	147/45	Intravenous infusion of trastuzumab deruxtecan at a dose of 6.4 mg per kilogram of body weight every 3 weeks	Intravenous infusion of irinotecan monotherapy at a dose of 150 mg per square meter of body-surface area administered every 2 weeks or paclitaxel monotherapy, at a dose of 80 mg per square meter administered on days 1, 8, and 15 every 4 weeks.	GC	OS,PFS,ORR,DCR,AE
Kohei Shitara (11)	III	RCT	337/170	64/63	369/138	Oral trifluridine/tipiracil (35 mg/m² twice daily on days 1–5 and days 8–12 every 28 days) plus best supportive care	Oral placebo plus best supportive care	GC/GEJC	OS,PFS,ORR,DCR,AE
Zhi Peng (15)	II	Non-RCT	125	58	91/34	RC48 2.5 mg/kg Q14d	/	GC/GEJC	OS,PFS,ORR,DCR,AE

### Integrated analysis of median overall survival

We integrated and analyzed the mOS of the same treatment regimen from different RCT studies to obtain the pooled OS (pOS) of the currently available third-line treatment. The pOS of apatinib and ADC as third-line treatments for GC/GEJC were 5.59 months (95% CI 3.96–7.21) and 10.12 months (95% CI 5.61–14.62), respectively (**Supplementary Figure 4**).

### Overall outcomes

**Figure 2:**
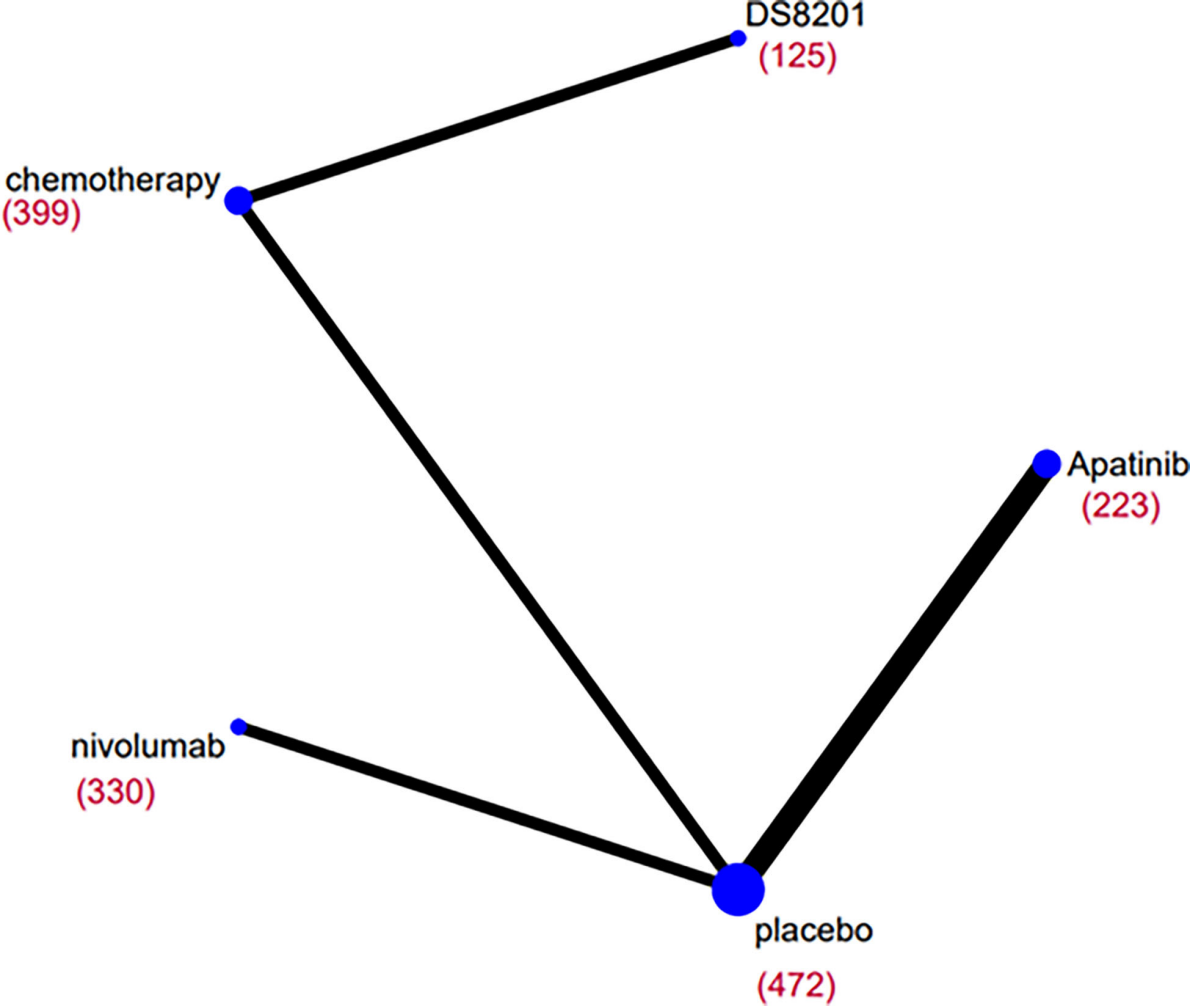
Network diagrams of comparisons on overall survival (OS) of treatments included in the network meta-analysis of the third-line treatments for advanced GC/GEJC. Each circular node represents a type of treatment. The size of the nodes and the thickness of the lines are weighted according to the number of studies evaluating each treatment and direct comparison, respectively. The total number of patients receiving treatments is shown in brackets.

**Figure 3:**
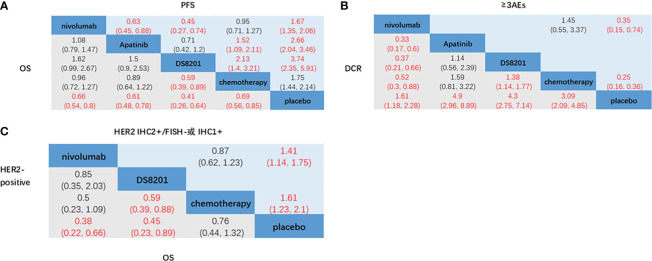
Network meta-analysis of the third-line treatments for advanced GC/GEJC. **(A)** Pooled hazard ratio (HR) [95% CrIs (credible intervals)] for overall survival (OS) and progression-free survival (PFS) in the overall population. **(B)** Pooled relative risk (RR) (95% CrIs) for disease control rate (DCR) and adverse events of grade 3 or higher (≥3AEs) in the overall population. **(C)** Pooled HR (95% CrI) for OS of patients with HER2-positive and HER2 IHC2+/FISH- or IHC1+.

The relative efficacy between these treatments was compared first, and the network diagram of direct and indirect comparisons of all treatment regimens are presented in **Figure 2**. In terms of OS (**Figure 3A**), nivolumab (HR: 0.66, 95% CI: 0.54-0.8) apatinib (HR: 0.61, 95% CI: 0.48-0.78), DS-8201 (HR: 0.41, 95% CI: 0.26-0.64), and chemotherapy (HR: 0.69, 95% CI: 0.56-0.85) were all significantly increased compared with that of placebo. DS-8201 prolonged the OS of patients significantly (HR: 0.59, 95% CI: 0.39-0.89) over chemotherapy. The SUCRA value of DS-8201 (0.98) was the largest in OS, indicating that it most likely ranked first, followed by apatinib (0.63) and nivolumab (0.49) (**Supplementary Figure 1A**). The PFS of placebo was significantly shorter than that of nivolumab (HR: 1.67, 95% CI:1.35-2.06), apatinib (HR: 2.66, 95% CI: 2.04-3.46), DS-8201 (HR: 3.47, 95% CI: 2.35-5.91), and chemotherapy (HR: 1.75, 95% CI: 1.44-2.14). Furthermore, the PFS of chemotherapy was shorter than that of apatinib (HR: 1.52, 95% CI: 1.09-2.11) and DS-8201 (HR: 2.13, 95% CI: 1.4-3.21) (**Figure 3A**). The SUCRA value of DS-8201 (0.97) was higher than that of apatinib (0.77) and chemotherapy (0.41) in PFS. For DCR (**Figure 3B**), nivolumab (RR: 1.61, 95% CI: 1.18-2.28), apatinib (RR: 4.9, 95% CI: 2.96-8.89), DS-8201 (RR: 4.3, 95% CI: 2.75-7.14), and chemotherapy (RR: 3.09, 95% CI: 2.09-4.85) were shown to have significantly better efficacy over placebo. In addition, apatinib (RR: 3.04, 95% CI: 1.65-5.95), DS-8201 (RR: 2.67, 95% CI: 1.51-4.83) and chemotherapy (RR: 1.92, 95% CI: 1.14-3.31) were superior to nivolumab. The SUCRA value for apatinib (0.89) was the largest in DCR, followed by DS-8201 (0.84). In terms of ≥3 AEs (**Figure 3B**), nivolumab (RR: 2.82, 95% CI: 1.36-6.86) and chemotherapy (RR: 4.07, 95% CI: 2.78-6.31) were associated with higher incidence rates of adverse events than placebo. According to the statistics of the incidence of ≥3 AEs for various treatments, hypertension(5.4%) and hand-foot syndrome(7.6%) were the most common adverse events for apatinib, while the incidence of hematological toxicity and gastrointestinal-related adverse events was low. As for DS-8201, the incidence of leukopenia (21%) and anemia (38%) were relatively high. (**Supplementary Figures 3**)

### NMA of different HER-2 expression status subgroup

According to the HER2 expression status of advanced GC patients, NCCN guidelines define HER2-overexpression as IHC2+ and IHC3+, in which IHC3+ and IHC2+/FISH+ are HER2-positive and IHC1+ is HER2-negative (6). Nivolumab (HR: 0.38, 95% CI: 0.22-0.66) and DS-8201 (HR: 0.45, 95% CI: 0.23-0.89) achieved significant OS benefits in patients who were HER2-positive compared to placebo. Furthermore, DS-8201 (HR: 0.59, 95% CI: 0.39-0.88) significantly prolonged the OS of patients compared with chemotherapy (**Figure 3C**). The SUCRA values of DS-8201 (0.85) and nivolumab (0.8) were significantly higher than that of chemotherapy (0.29) in HER2-positive patients (**Supplementary** **Figure 1**). For patients with HER2 IHC2+/FISH- or IHC1+, the OS of placebo was shorter than that of nivolumab (HR: 1.41, 95% CI: 1.14-1.75) and chemotherapy (HR: 1.61, 95% CI: 1.23-2.1). However, the SUCRA value of nivolumab (0.61) was still higher than that of chemotherapy (0.39). Because Destiny-Gastronomy 01 (2020) (12) did not document the HR values of patients who were HER2-positive (IHC2+/FISH-) and HER2-negative (IHC1+), we cannot further compare the relative efficacy of DS-8201 with other treatments. However, the results of the exploratory cohort study of DS-8201 by DESTINY-Gastric01 showed that the mOS of HER2-positive, IHC2+/FISH-, and IHC1+ were 12.6 months (95% CI: 0.4-33.2), 7.8 months (95% CI: 0.2-27.7) and 8.5 months(95% CI: 1.8-23.1) respectively, all meeting the primary endpoint of OS.

### NMA of histopathology subgroup

For GC patients with different histopathology, nivolumab (HR: 0.62, 95% CI: 0.44-0.87), DS-8201 (HR: 0.38, 95% CI: 0.2-0.72), and chemotherapy (HR: 0.58, 95% CI: 0.39-0.86) all effectively prolonged the OS of patients with the intestinal type of GC over placebo, but no significant differences among these treatments were found. For patients with the diffuse type of GC, the OS of chemotherapy (HR: 2.63, 95% CI: 1.18-5.9) and placebo (HR: 3.82, 95% CI: 1.36-10.81) were significantly shorter than that of DS-8201. However, nivolumab did not achieve OS benefits over chemotherapy and placebo (**Supplementary Figure 5A**). The SUCRA values of DS-8201 was the largest for both patients with the intestinal type of GC (0.95) and patients with the diffuse type of GC (0.98) (**Supplementary Figure 1**).

### NMA of previous gastrectomy, primary sites subgroup

For patients with a gastrectomy history, nivolumab (HR: 0.61, 95% CI: 0.47-0.78), DS-8201 (HR: 0.09, 95% CI: 0.03-0.3), and chemotherapy (HR: 0.57, 95% CI: 0.41-0.79) were superior to placebo. Furthermore, DS-8201 significantly prolonged the OS of patients compared with chemotherapy (HR: 0.16, 95% CI: 0.05-0.49) and nivolumab (HR: 0.15, 95% CI: 0.05-0.5). However, only nivolumab (HR: 0.71, 95% CI: 0.51-0.99) achieved OS benefits for patients in the absence of gastrectomy compared with placebo (**Supplementary Figure 5B**). Nivolumab (HR: 0.69, 95% CI: 0.56-0.86), DS-8201 (HR: 0.4, 95% CI: 0.24-0.66), and chemotherapy (HR: 0.67, 95% CI: 0.52-0.86) were superior to placebo in patients with gastric originated cancer. DS-8201 treatment showed significant OS benefits compared with chemotherapy (HR: 0.59, 95% CI: 0.38-0.92). However, only nivolumab (HR: 0.42, 95% CI: 0.2-0.89) prolonged the OS of patients with GEJC over placebo (**Supplementary Figure 5C**).

### NMA of age, gender, ECOG and region subgroup

Nivolumab (HR: 0.6, 95% CI: 0.44-0.82) and DS-8201 (HR: 0.32, 95% CI: 0.17-0.61) significantly prolonged the OS of patients over 65 years old comparing with placebo. In addition, DS-8201 (HR: 0.44, 95% CI: 0.26-0.75) was superior to chemotherapy. For patients less than 65 years old, nivolumab (HR: 0.7, 95% CI: 0.54-0.91) and chemotherapy (HR: 0.67, 95% CI: 0.51-0.89) achieved OS benefits over placebo (**Supplementary Figure 5D**). For males, the OS of nivolumab (HR: 0.6, 95% CI: 0.48-0.76), DS-8201 (HR: 0.34, 95% CI: 0.2-0.6), and chemotherapy (HR 0.65, 95% CI 0.5-0.84) were longer than that of placebo. However, DS-8201 was still superior to chemotherapy (HR: 0.53, 95% CI: 0.33-0.86). No third-line treatment had a significant effect on female patients (**Supplementary Figure 5E**). Nivolumab (HR: 0.62, 95% CI: 0.43-0.89), DS-8201(HR 0.38, 95% CI 0.19-0.75), and chemotherapy (HR 0.67, 95% CI 0.47-0.96) prolonged the OS of patients with ECOG PS=0 compared with that of placebo, which was the same as for patients with ECOG PS=1. Furthermore, DS-8201 can effectively improve the OS of patients over apatinib (HR: 0.49, 95% CI: 0.24-0.99) (**Supplementary Figure 5F**). The SUCRA values of DS-8201 was the largest for both patients with ECOG PS=0 (0.9) and patients with ECOG PS=1 (0.97). The results of subgroup analysis on Asian patients showed that nivolumab (HR: 0.46, 95% CI: 0.23-0.92), apatinib (HR: 0.71, 95% CI: 0.54-0.94), and DS-8201 (HR: 0.44, 95% CI: 0.22-0.86) prolonged the OS of patients significantly over placebo. However, DS-8201 still showed significant OS benefits over chemotherapy (HR: 0.57, 95% CI: 0.36-0.89) (**Supplementary Figure 5G**).

### NMA of number of previous regimen treatments and metastasis sites subgroup

For patients with two previous lines of treatment, apatinib (HR: 0.7, 95% CI: 0.49-0.99) and chemotherapy (HR: 0.68, 95% CI: 0.47-0.97) were superior to placebo. Notably, DS-8201 improved the overall survival (OS) of patients with three previous lines of treatment compared with nivolumab (HR: 0.37, 95% CI: 0.15-0.9), apatinib (HR: 0.38, 95% CI: 0.14-0.99), chemotherapy (HR: 0.39, 95% CI: 0.18-0.85), and placebo (HR: 0.29, 95% CI: 0.12-0.68), while apatinib failed to achieve survival benefits (**Supplementary Figure 5H**). For patients with two or less metastasis sites, apatinib (HR: 0.7, 95% CI: 0.51-0.97), DS-8201 (HR: 0.27, 95% CI: 0.09-0.88) and chemotherapy (HR: 0.68, 95% CI: 0.49-0.95) were superior comparing with placebo. However, more than two metastasis sites were observed and apatinib failed to improve the OS of patients. Additionally, DS-8201 was superior to chemotherapy (HR 0.61, 95% CI 0.39-0.95) (**Supplementary Figure 5I**).

### Rank probabilities

Bayesian ranking curves for various treatment options in different subgroups of patients are shown in (**Supplementary Figures 1, 2**). The results of Bayesian ranking were consistent with NMA approximately. For the overall population, ADC ranked first in both OS (0.98) and PFS (0.97), followed by apatinib and nivolumab. Apatinib ranked first in DCR (0.89). In addition, ADC ranked first in patients with a HER2-positive diagnosis, intestinal/diffuse histopathology, with or without previous gastrectomy history, gastric origination cancer, and ECOG PS=0/1, as well as in patient subgroups with two or more previous regimens, any numbers of metastasis site, and Asian patients. ADC was followed by nivolumab and apatinib. For patients with GEJC, nivolumab ranked first.

### Model convergence, assessment of risk of bias, analysis of heterogeneity and inconsistency

As shown in **Supplementary Figure 6**, the results of the risk of bias assessment indicated low risk of bias for most RCTs and that non-RCTs were high-quality studies. The trace plots and Brooks-Gelman-Rubin diagnostics showed great convergence of the models we used (**Supplementary Figure 7**). In addition, the consistent model showed similar or better degree of fit than the inconsistent model in most of the comparisons (**Supplementary Table 3**). The heterogeneity between the available RCTs was large (I^2^>50%) for primary outcomes. We performed a meta-analysis showing that JAVELIN Gastric300 (2018) (24) had a great influence on the heterogeneity of the NMA. No significant differences were found in the study design, median age of patients, or the ratio of male to female patients. In addition, factors such as age, sex, race, ECOG PS, primary sites, and PD-L1 expression status of patients in JAVELIN Gastric300 (2018) were analyzed in subgroups which showed that the statistical heterogeneity of each subgroup was low or medium (figure). Thus, we suggested that the heterogeneity between JAVELIN Gastric300 (2018) and other RCTs had little to do with the baseline characteristics of the patients. In addition, methodological heterogeneity was excluded because JAVELIN Gastric300 (2018) followed the principles of distribution concealment and blindness. Furthermore, the results of JAVELIN Gastric 300 suggested that the third-line treatment of GC/GEJC patients using the single drug avelumab did not lead to an improvement in OS or PFS over chemotherapy. Therefore, we excluded this RCT. In total, we included five RCTs with low statistical heterogeneity (I^2^<50%) (**Supplementary Figure 8**).

## Discussion

Combination therapy with anti-tumor drugs can prolong the OS of patients with GC/GEJC and lead to the improvement of patients’ quality of life. Presently, third or later-line treatment options for advanced GC patients recommended by CSCO guidelines include immunotherapy (nivolumab), chemotherapy(irinotecan, FTD/TPI) and targeted therapy(apatinib) (7). For HER2-positive (IHC3+ or IHC2+/FISH+) patients, the guidelines recommended the use of ADC (trastuzumab deruxtecan, DS-8201) and virtuximab (RC48-ADC). However, in the phase III ATTRACTION-2 trial, regardless of HER2 expression status, nivolumab significantly prolonged the OS of unresectable advanced or recurrent GC/GEJC (5.3 months vs 4.1 months) and reduced the risk of death (HR: 0.62, 95% CI: 0.50-0.75) (22) over placebo. T-DXd/DS8201 not only achieved significant survival benefits in GC patients who were HER2-positive, but also showed clinical activity in patients who were HER2-negative (IHC1+) or had low HER2 expression(IHC2+/FISH-) in the exploratory subgroup analysis of DESTINY-Gastric01(2020) (12). The results showed that the mPFS and mOS of the low-expression group were 4.4 months (95% CI: 2.7-7.1) and 7.8 months (95% CI: 4.7 -NE) respectively. The mPFS and mOS were 2.8 months (95% CI: 1.5-4.3) and 8.5 months (95% CI: 4.3-10.9), respectively, for the HER2-positive group. The median DOR of the two groups were 4.2 months (95% CI: 1.2-10.5) and 2.8 months (95% CI: 0.7-14.9), respectively. To further accurately screen and optimize the third-line treatment options through systematic review and NMA, the efficacy and safety of published third-line treatments for advanced GC/GEJC were reviewed to provide guide in selecting the best third-line treatment, so as to maximize precise individualized treatment plans for advanced GC/GEJC.

As shown in ATTRACTION-2 (22), Jinli (2016) (10)and Kohei Shitara (2018) (11), the OS of nivolumab (HR: 0.66, 95% CI: 0.54-0.8), apatinib (HR: 0.61, 95% CI: 0.48-0.78), and chemotherapy(HR: 0.69, 95% CI: 0.56-0.85) met the expected endpoint comparing with placebo in the third-line treatment of advanced GC/GEJC, with a mOS of 5.26 months (95% CI: 4.6-6.37), 6.5 months (95% CI: 4.8-7.6), and 5.7 months (95% CI: 4.8-6.2) respectively. In addition, nivolumab (RR: 1.61, 95% CI: 1.18-2.28), apatinib (RR: 4.9, 95% CI: 2.96-8.89), and chemotherapy (RR: 3.09, 95% CI: 2.09-4.85)were all superior to placebo, with a DCR of 40.2%, 42.0%, and 44.1% respectively. With the advent of ADC (DS-8201/RC48), which had been approved for the third-line treatment of GC/GEJC (15), the results of DESTINY-Gastric01 (2020) (12)showed that DS-8201 significantly prolonged the OS of patients in comparison with chemotherapy (HR: 0.59, 95% CI: 0.39–0.88), with mOS of 12.5 months (95% CI: 9.6–14.3). The DCR of DS-8201 was 85.7%, which was significantly improved comparing with that of chemotherapy (RR: 4.3, 95% CI: 2.75-7.14). This shows how the third-line treatment options of advanced GC continue to develop. The result of statistical heterogeneity test showed that the heterogeneity among RCTs we included was low (I2 < 50%), thus they were comparable. We then performed a NMA to compare the survival benefits of each third-line treatment option which showed that the SUCRA values of nivolumab (0.49) and apatinib (0.63) were higher than that of chemotherapy (0.41) in the general population, indicating that nivolumab and apatinib were superior to chemotherapy. This was consistent with the NMA results of third-line treatments of advanced GC/GEJC conducted by Park et al (25) and Huang et al (26). On this basis, the results of our NMA showed that the SUCRA value of DS-8201 (0.98) was the largest in OS. Furthermore, the NMA results of DCR showed that the SUCRA values of DS-8201 (0.84) and apatinib (0.89) were significantly higher than that of chemotherapy (0.52). DESTINY-Gastric01 showed that the most common ≥3AEs were the decreased neutrophil count (in 51% of the patients in the trastuzumab DS-8201 group), anemia (in 38% of the patients) and decreased white-cell count (in 21% of the patients). One death in the trastuzumab DS8201 group was considered by the investigators to be related to therapy, due to pneumonia, so it was concluded that its safety was controllable. In brief, we suggest that ADC is the best third-line treatment for advanced GC for the general population, followed by apatinib.

The overexpression of HER2 has been identified as a predictive biomarker in advanced GC, including IHC2+ and IHC3+, in which IHC3+, IHC2+/FISH+ are HER2-positive, while IHC1+ is HER2-negative (6). In a randomized, multicenter, phase 3 ToGA trial of Trastuzumab for Gastric Cancer, all patients for potential enrollment were tested for HER2 expression by IHC and FISH. The results showed that the ratio of HER2-positive IHC2+/FISH- was 17.8% and 5.3% (27). The first-line treatment of HER2-positive advanced GC with a combination of Trastuzumab with chemotherapy exhibited a median OS of 13.8 months and had efficacy outcomes correlated with the level of HER2 expression. In recent years, ADC (DS-8201, RC48) has offered a remarkable option in the third-line treatment for advanced GC patients with HER2-overexpression. The results of exploratory cohort study of DS-8201 by DESTINY-Gastric01 showed that the mPFS and mOS of the HER2-positive group were 5.6 months (95% CI: 4.3-6.9) and 12.6 months (95% CI: 0.4-33.2) respectively, compared with 4.4 months (95% CI: 2.7-7.1) and 7.8 months (95% CI: 0.2-27.7) for the IHC2+/FISH- group respectively, and 2.8 months (95% CI: 1.5-4.3) and 8.5 months (95% CI: 1.8-8) for HER2-negetive group respectively. A single-arm phase II study (2021) (15) showed that the mPFS and mOS in the RC48 group were 4.1 months (95% CI: 3.7-4.9) and 7.9 months (95% CI: 6.7-9.9), respectively. In addition, the exploratory subgroup analysis of ATTRACTION-2 compared the efficacy of nivolumab for patients with different HER2 expression statuses, and the results showed that the OS of patients with prior trastuzumab use was significantly longer comparing with that of placebo (HR: 0.38 95% CI: 0.22–0.66) which met the expected endpoint of OS. The mPFS, mOS, and median DOR were 1.6 months (95%CI: 1.5-4), 8.3 months (95% CI: 5.3-11) and 8.6 months (95% CI: 4.3-13.1) respectively. Similarly, for patients without prior trastuzumab use, nivolumab achieved OS benefits comparing with placebo (HR: 0.71, 95% CI: 0.57–0.88), and the mPFS, mOS, and median DOR were 1.6 months (95% CI: 1.5-2.4), 4.8 months (95% CI: 4.1-6), and 9.5 months (95% CI: 2.8-22.9)respectively. This result indicates that nivolumab was effective as a third or later-line treatment for GC/GEJC regardless of prior trastuzumab use (28) or HER2 expression status, and that nivolumab could benefit the survival of GC/GEJC patients comparing with placebo. We compared the relative efficacy of ADC, nivolumab, and chemotherapy in patients with different HER2 expression statuses based on NMA, and the results showed that the SUCRA value of DS-8201 (0.85) and nivolumab (0.8) were significantly higher than that of chemotherapy (0.29) in HER2-positive patients. For patients with HER2 (IHC2+/FISH- or IHC1+), the SUCRA value of nivolumab (0.61) was still higher than that of chemotherapy (0.39). Since DESTINY-Gastric01(2020) did not record the HR value of OS in patients who were HER2-positive (IHC2+/FISH-) or HER2-negative (IHC1+), it was impossible to further compare the relative efficacy of DS-8201 with other treatments. We also examined the reason why ADC (DS-8201, RC48) was effective as a third-line treatment for GC patients with HER2 overexpression. A derivative of DX-8951f (DXd), a topoisomerase I inhibitor, is coupled to the anti-HER2 antibody *via* a peptide (GGFG) linker. This stable linker is cleaved upon internalization by lysosomal enzymes such as cathepsin B and L, which are highly expressed in tumor cells (29–31). As a result, ADC can be internalized into tumor cells *via* the HER2 receptor and cleaved by lysosomal enzymes, releasing DXd to specifically attack target molecules in tumor cells (32) which might be particularly effective in the treatment of tumors with overexpression or heterogeneous expression of HER2 (12).

In addition, our study also completed NMA of other subgroups, based on different demographic characteristics and pathological types. The results showed that for patients with intestinal type of GC, the SUCRA values of DS-8201, nivolumab, and chemotherapy were 0.95, 0.5, and 0.54, respectively. For patients with a gastrectomy history, the values were 0.99, 0.46, and 0.54, respectively, and for patients with gastric origination cancer, the values were 0.99, 0.48, and 0.53 respectively. The SUCRA ranking of patients with an ECOG PS score of 0-1 was the same as above, indicating that DS-8201 was significantly superior to chemotherapy, and may be the best third-line treatment for these patient groups, followed by nivolumab. For patients with GEJC, the results of ATTRACTION−2 (22) showed that nivolumab (HR: 0.42, 95% CI: 0.2–0.89) prolonged the OS of patients over placebo. Our NMA results showed that the SUCRA value of nivolumab (0.83) was significantly higher than that of DS-8201(0.66) and chemotherapy (0.42), indicating that nivolumab was the best third-line treatment option for patients with GEJC, which agreed with the results of subgroup analysis by Huang et al (26). Previous studies have demonstrated that GC in Western populations tends to originate mainly from GEJ (33). The pathological type of GEJC that includes a portion of squamous cell carcinoma tends to respond better to immunotherapy compared with adenocarcinoma. These factors might produce the more beneficial outcome of OS for GEJC from nivolumab (26). Notably, the results of ATTRACTION−2 (22), Jinli (2016), Kohei Shitara (2018), and DESTINY-Gastric01 showed that nivolumab (HR: 1.26, 95% CI: 0.87–1.83), DS-8201 (HR: 1.55, 95% CI: 0.66-3.71), and chemotherapy (HR: 1.22, 95% CI: 0.79-1.88) did not significantly prolong the OS of female patients over placebo, indicating that no third-line treatment had a significant effect on female patients. On the one hand, among the RCTs we included, there was less percentage of female patients than that of male patients, so the sample size was smaller, which may be related to the higher incidence of GC in male population (34). On the other hand, some studies have found that the pathological types of poorly differentiated adenocarcinoma and signet ring cell carcinoma were more frequently observed female GC patients than in male GC patients, and that these pathological types of tumor cells respond rather poorly to anticancer therapy (35, 36), thus leading to this result. In brief, we suggest that ADC is the best third-line treatment for the overall population of GC, as well as for patient groups with HER2-overexpression, intestinal histopathology, previous gastrectomy history, gastric origination cancer, and ECOG PS=0/1, followed by nivolumab and apatinib. However, nivolumab is the best third-line treatment for patients with GEJC.

The present study had the following limitations. Firstly, because of the limited number of clinical trials of third or later-line treatments for patients with GC/GEJC, the number of studies and patients we included were limited. As a result, the conclusions of NMA need to be further verified. Secondly, the node analysis using the Bayesian method or the direct element analysis using the frequency method was not carried out, because a closed loop in our NMA could not be established. Therefore, we cannot evaluate the analysis inconsistency caused by heterogeneity (37). In addition, the results of SUCRA ranking did not directly reflect the superiority of treatment regimens, and when SUCRA predictions were inconsistent with NMA results, we preferentially made judgments based on the HR of NMA and its 95% CI. For the original study took GC and GEJC patients as a whole and did not further compare the efficacy of the drugs on GC and GEJC patients separately, we were not yet able to further compare the relative efficacy and safety of several therapeutic drugs on GC and GEJC separately and hope to conduct future clinical trials on GC or GEJC patients separately. In order to verify the reliability of the NMA conclusion, we hope to carry out more multi-center real-world research on third-line therapies of advanced GC/GEJC patients in the future and compare the relative efficacy of different intervention methods to provide a guide for the formulation of precise individualized treatment plans. In addition, we hope that future RCTs will further study tumor progression, tumor markers and clinical symptoms of adverse reactions so that the efficacy and safety of drugs can be more comprehensively evaluated.

## Conclusions

In summary, nivolumab, apatinib, chemotherapy, and ADC all improved the OS of GC/GEJC patients significantly. ADC may be the best third-line treatment option for the overall population of GC, as well as for patients with HER2-overexpression, intestinal histopathology, previous gastrectomy history, gastric origination cancer, ages over 65 and ECOG PS=0/1, followed by nivolumab and apatinib. Nivolumab may be the first treatment option for GEJC patients. For the limited clinical trials of third or later-line treatments for patients with GC/GEJC, these results need to be further confirmed by more multi-center real-world research in the future.

## Data availability statement

The original contributions presented in the study are included in the article/**Supplementary Material**. Further inquiries can be directed to the corresponding author.

## Author contributions

DY contributed to the study concept and design. YX and CZ participated in the initial literature search and evaluated the feasibility study for eligibility. YX and CZ interpreted the findings and wrote the first draft of the manuscript, prepared the figures and tables. JW contributed to language polishing. All authors contributed to the article and approved the submitted version.
