# Supplementary material for: Comparison of the efficacy and safety of third-line treatments for advanced gastric cancer: A systematic review and network meta-analysis
Source: Front Oncol. 2023 Mar 1;13:1118820. doi: 10.3389/fonc.2023.1118820 (PMC10016689; doi:10.3389/fonc.2023.1118820)
Supplement: Supplementary file 1 [file DataSheet_1.docx]

**
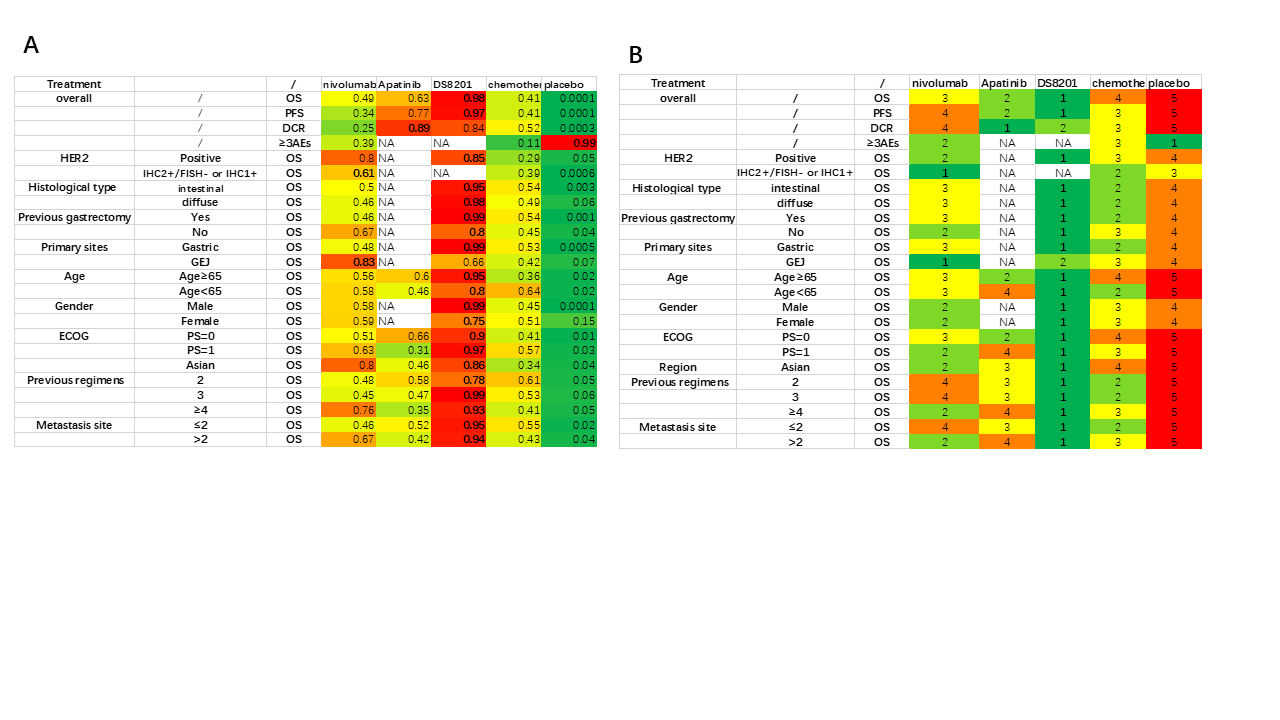
**

**Supplementary Figure 1**

**Bayesian ranking profiles of comparable treatments on efficacy and safety for patients with advanced GC/GEJC**. (A) Value of the surface under the cumulative ranking curve (SUCRA) in each cell indicates the probability of each treatment being ranked from first (high value) to last (low value) on overall survival (OS), OS for subgroups, progression free survival (PFS), DCR and ≥3AEs, the probability of value being ranked first are bold. (B) Number in each cell indicate the probability of each treatment being ranked from first to last on OS, PFS, DCR, ≥3AEs and OS subgroup according to the value of surface under the cumulative ranking curve (SUCRA).


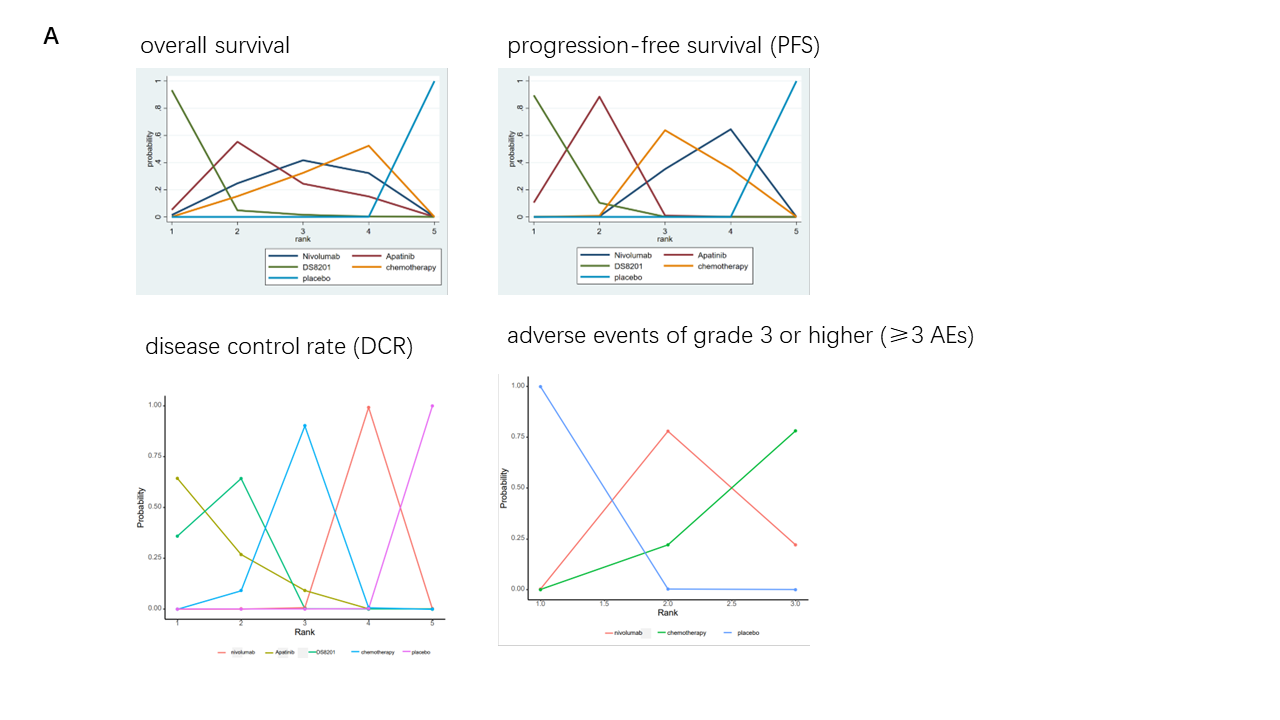

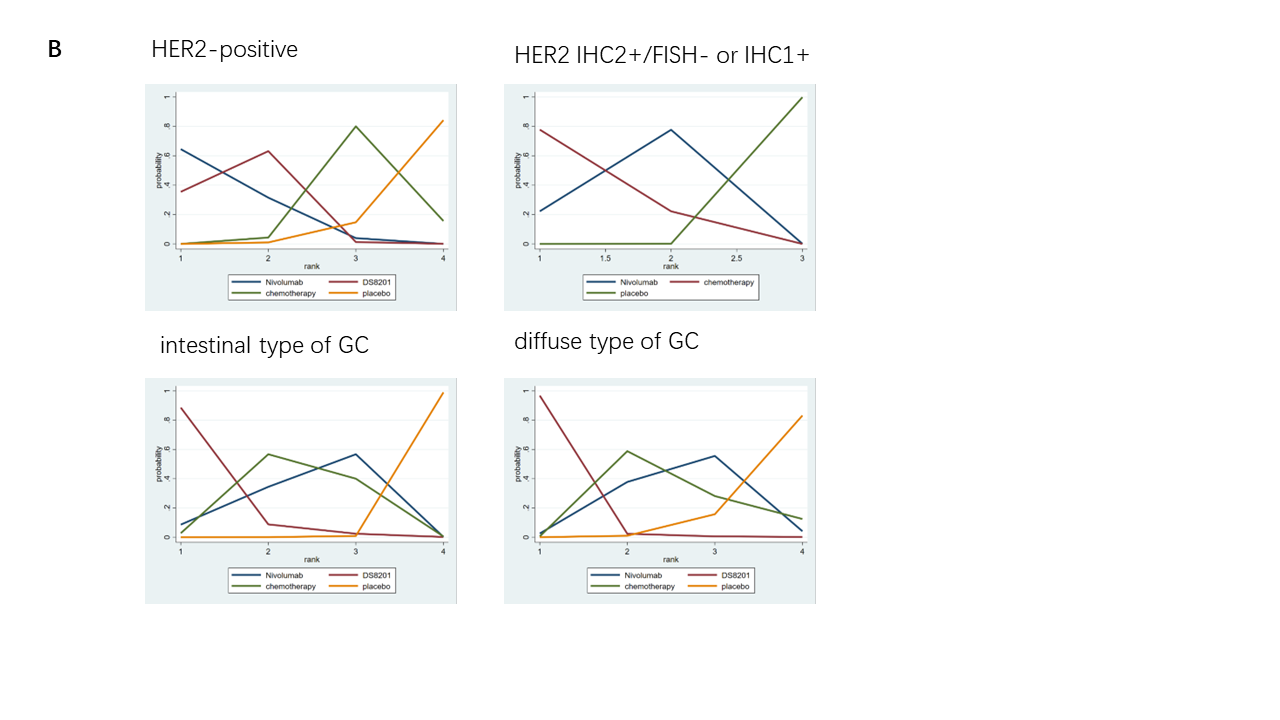

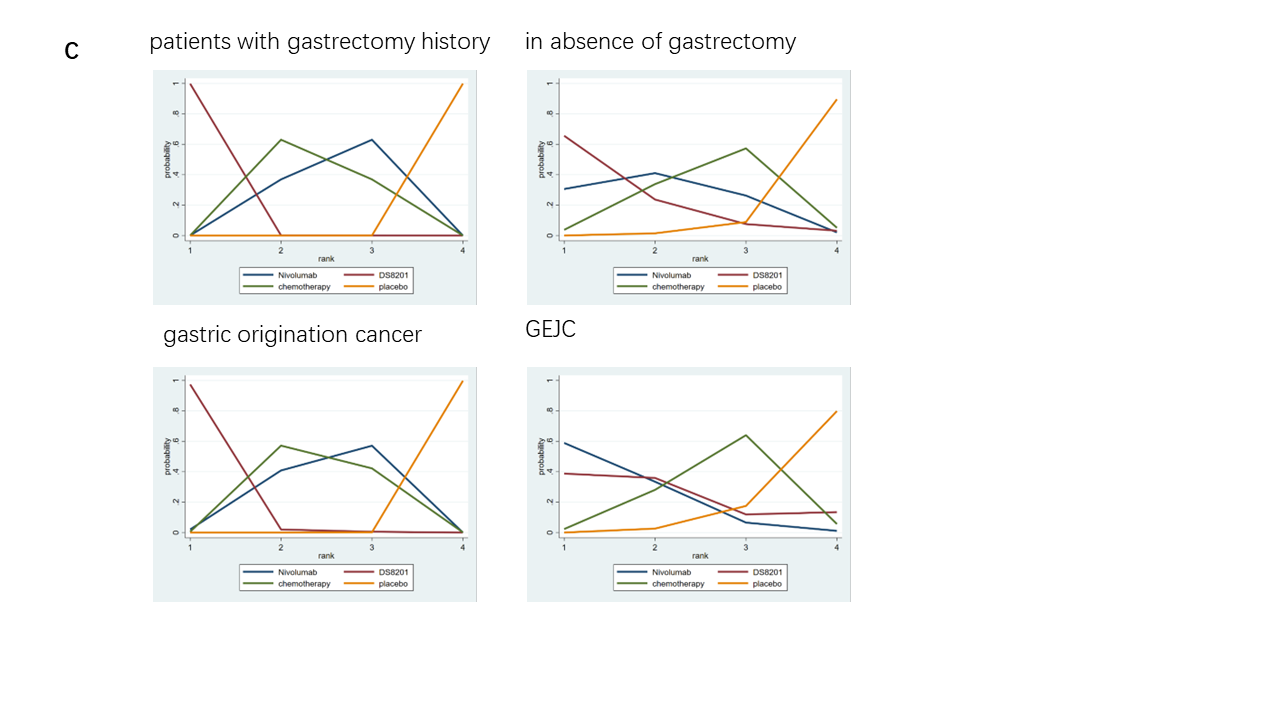

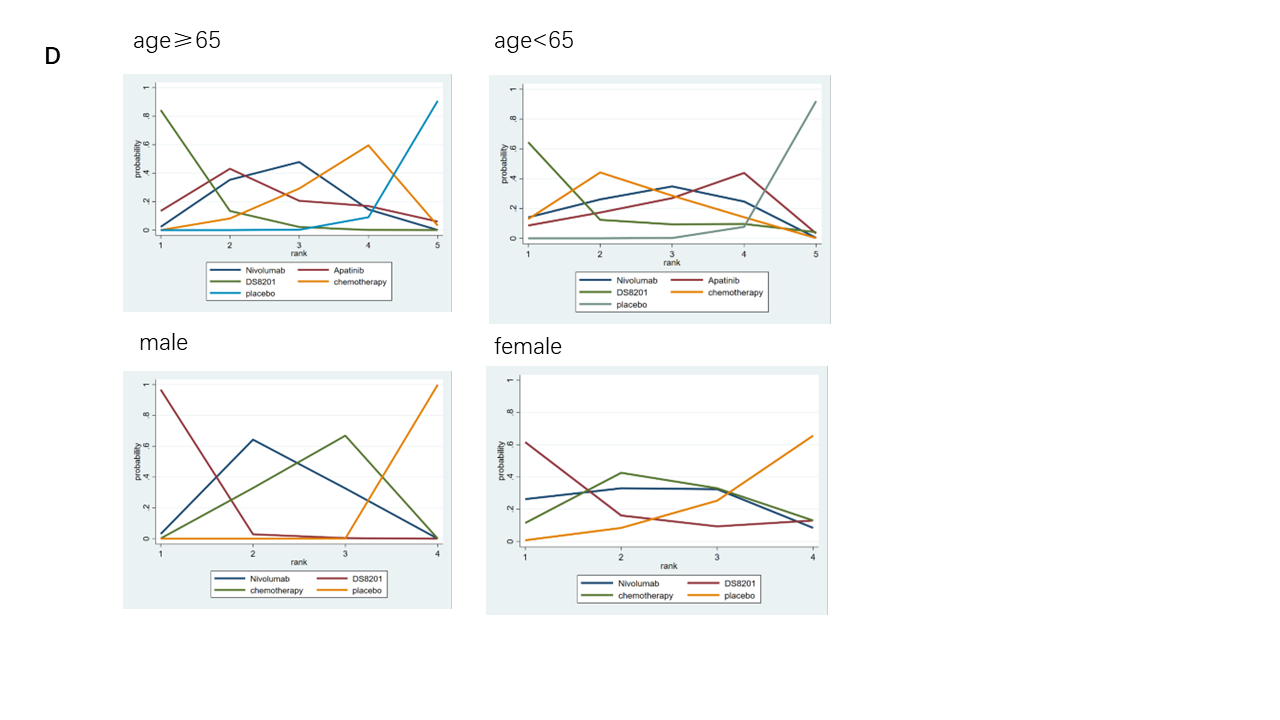

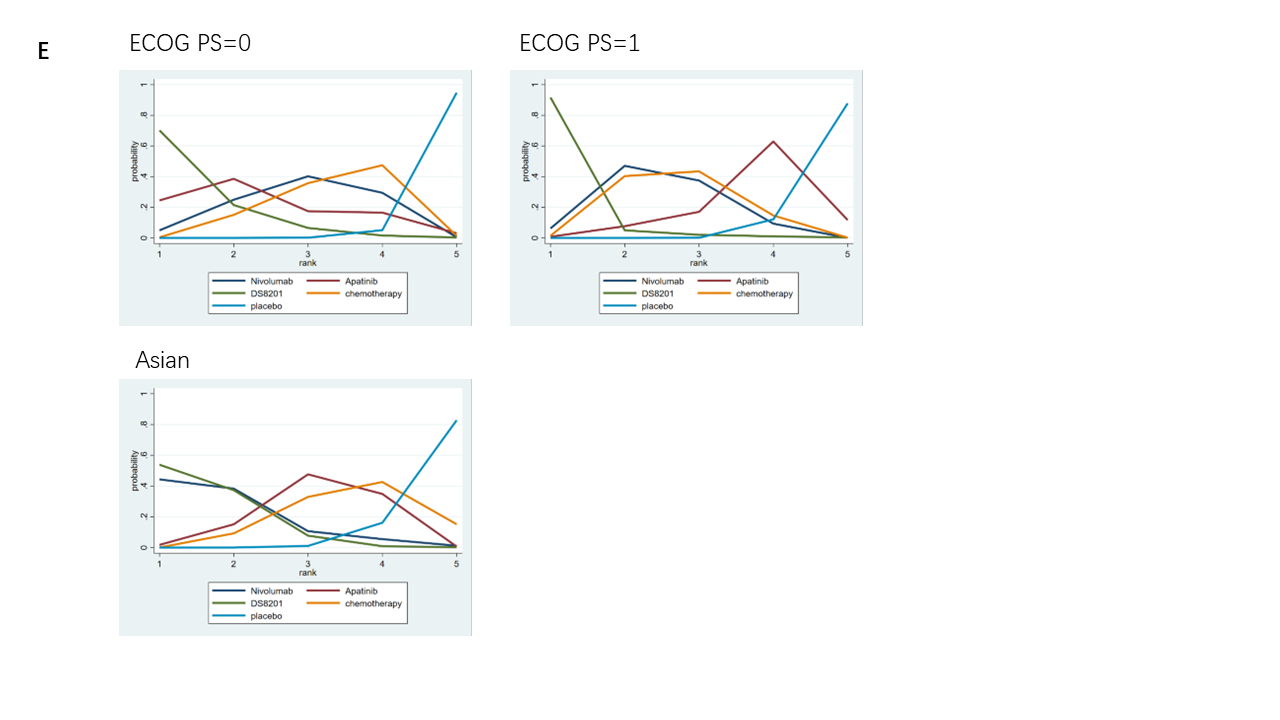

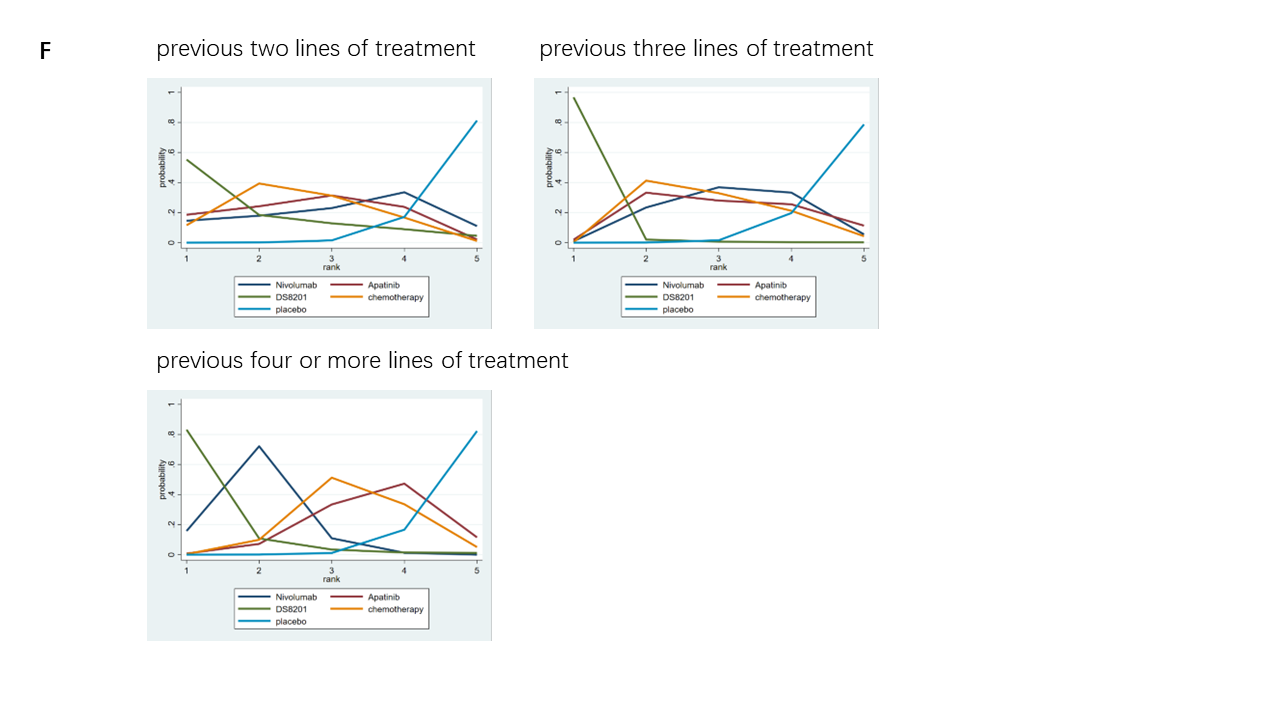

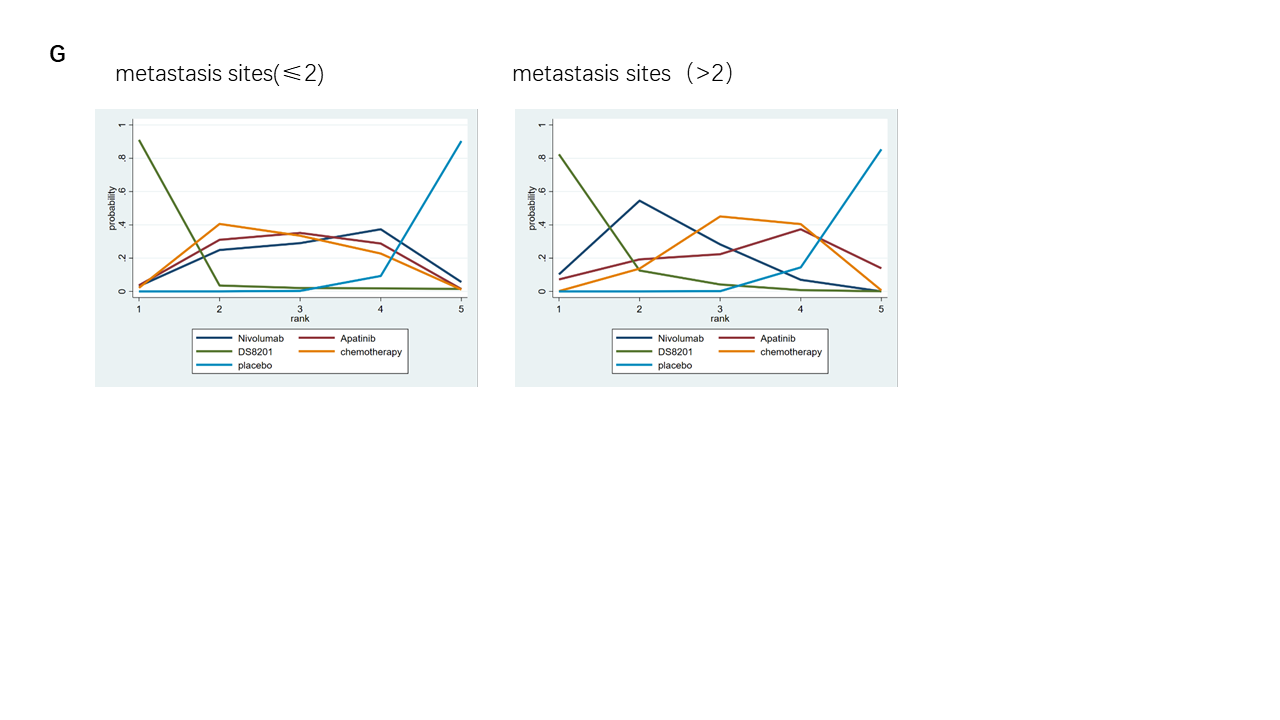


**Supplementary Figure 2**

**Bayesian ranking profiles of comparable treatments on efficacy and safety for patients with advanced GC/GEJ cancer.** Ranking curves indicating the probability of each comparable treatment being ranked from first to last. (A) overall survival (OS), progression-free survival (PFS), disease control rate (DCR), adverse events of grade 3 or higher (≥3 AEs).

(B) OS for HER2-positive, HER2 IHC2+/FISH- or IHC1+ subgroup, intestinal type of GC, diffuse type of GC subgroup (C) OS for patients with gastrectomy history, in absence of gastrectomy, gastric origination cancer, GEJC subgroup (D) OS for age≥65,

age<65, male, female subgroup (E) OS for ECOG PS=0, ECOG PS=1, Asian subgroup (F) OS for previous two, three, four or more lines of treatment subgroup (G) OS for metastasis sites(≤2) ，（>2） subgroup.


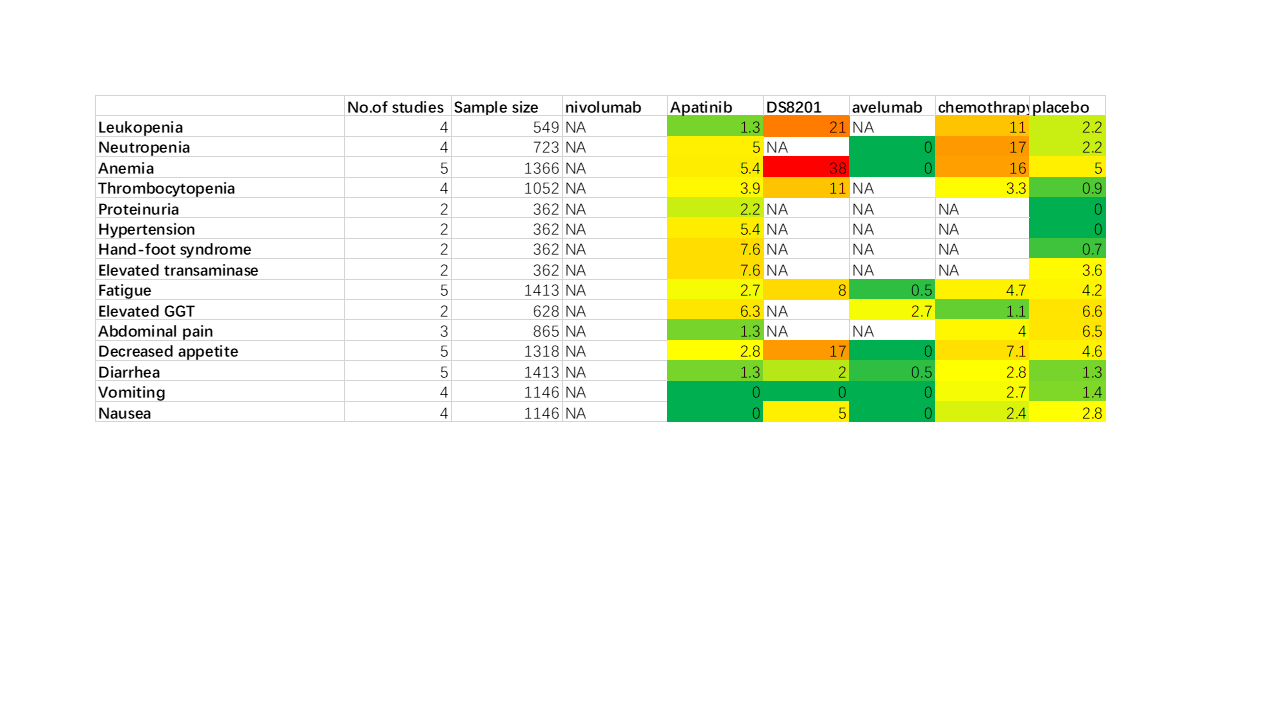
**Supplementary Figure 3**

A frequency toxicity profile in relation to the incidence (%) of each ≥3 AEs based on the population of each treatment in NMA we included.


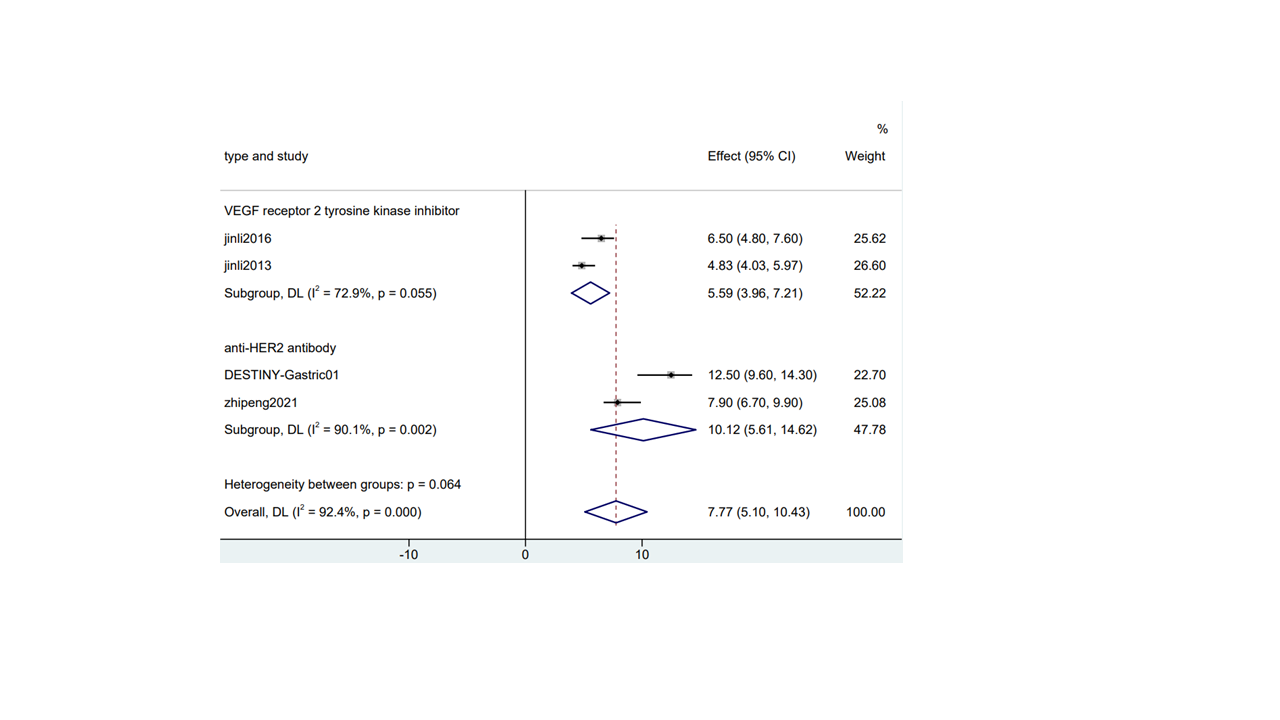


**Supplementary Figure 4**

Pooled median overall survival (POS) from integrated analysis of apatinib and ADC in patients with advanced GC/GEJC. GC/GEJC, Gastric cancer/Gastroesophageal junction cancer.


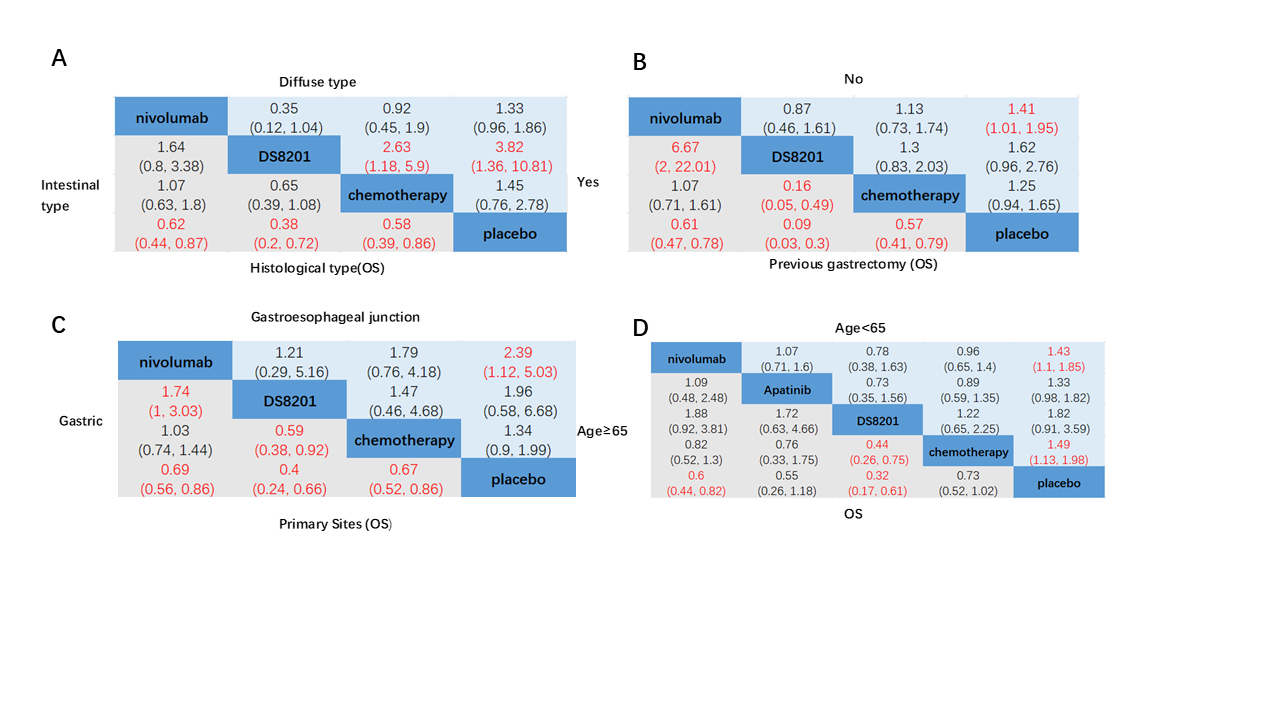

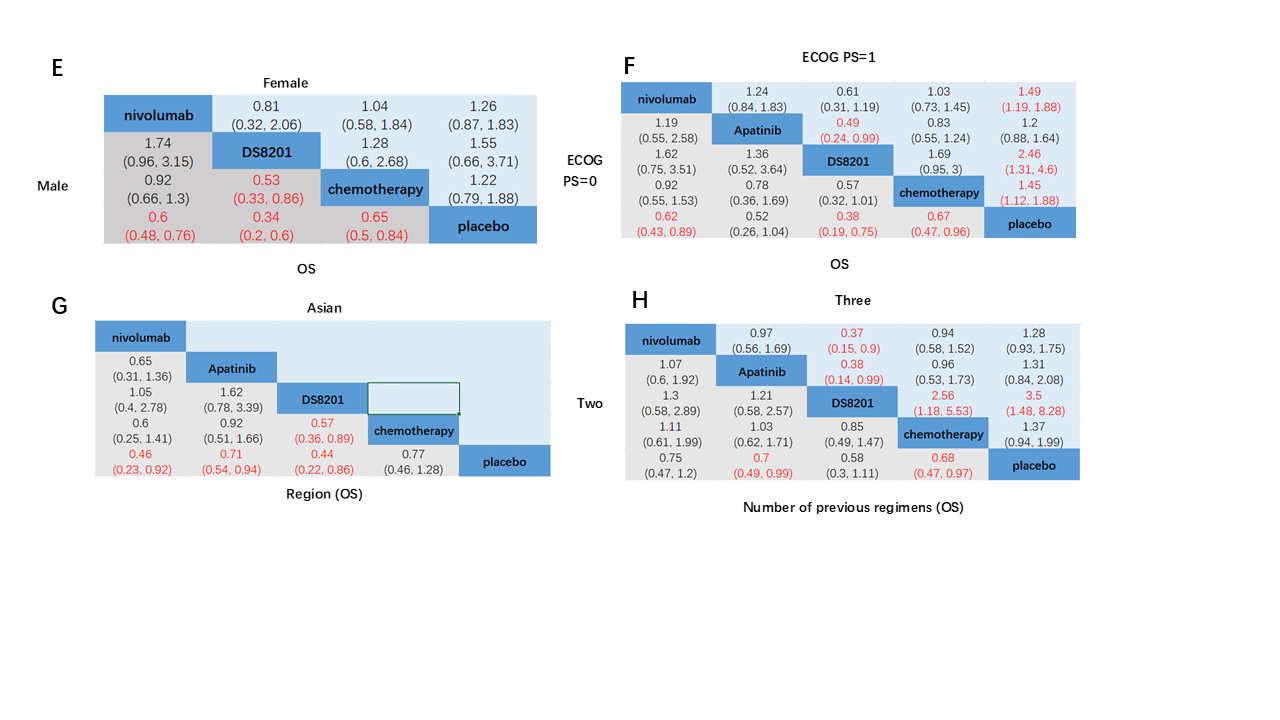

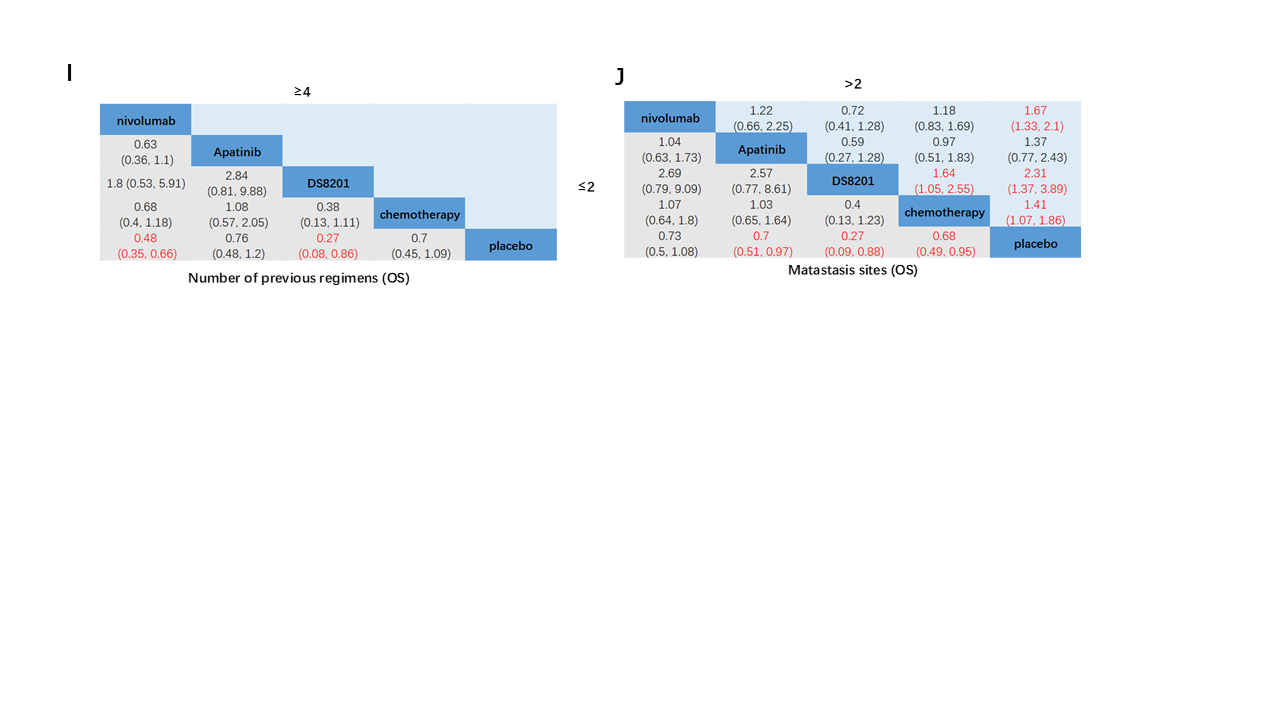


**Supplementary Figure 5**

Network meta-analysis of the third-line treatments for advanced GC/GEJC.

(A) Pooled HR (95% CI) for OS of histological type subgroup. (B) Pooled HR (95% CI) for OS of previous gastrectomy (Yes, No) (C) Pooled HR (95% CI) for OS of primary sites (Gastric, Gastroesophageal junction). (D) Pooled HR (95% CI) for OS of age ≥ 65 and < 65. (E) Pooled HR (95% CI) for OS of female and male. (F) Pooled HR (95% CI) for OS of Eastern Cooperative Oncology Group Performance Status = 0/1 (ECOG PS=0, ECOG PS=1). (G) Pooled HR (95% CI) for OS of Asian. (H) Pooled HR (95% CI) for (OS) of number of previous regimens (two, three). (I) Pooled HR (95% CI) for (OS) of number of previous regimens (four or above). (J) Pooled HR (95% CI) for OS of metastasis sites (two or less, three or more). HR less than 1 and RR more than 1 favor upper-row treatment. Significant results are highlighted in red


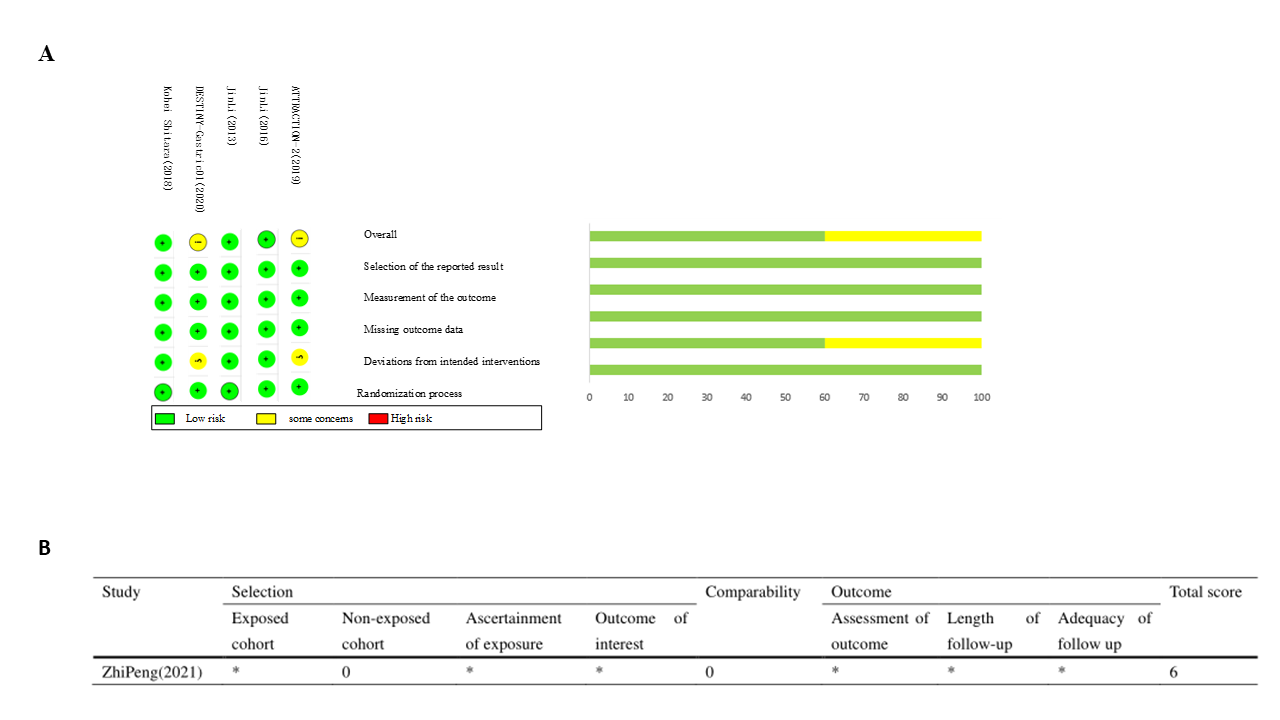


**Supplementary Figure 6**

1. Summary of results from bias risk assessment of RCTs using the Cochrane risk of bias tool.
2. Summary of results from bias risk assessment of non-RCT using the Newcastle-Ottawa scale.


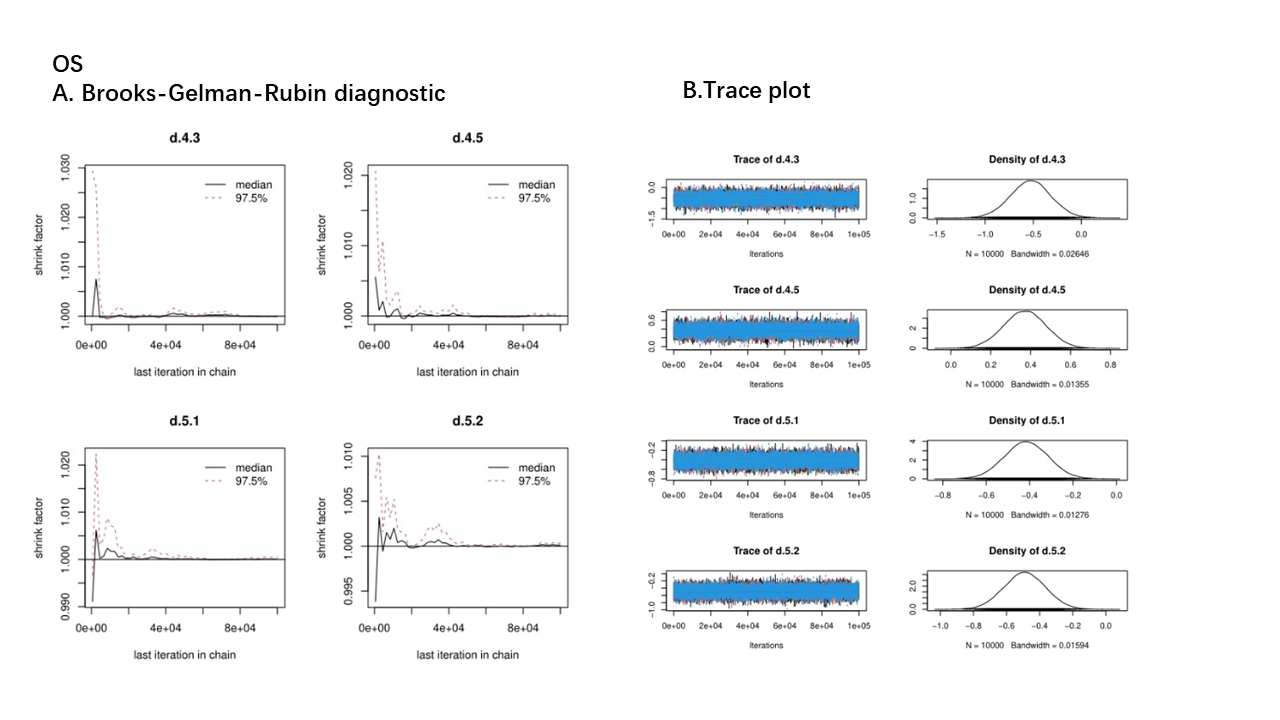

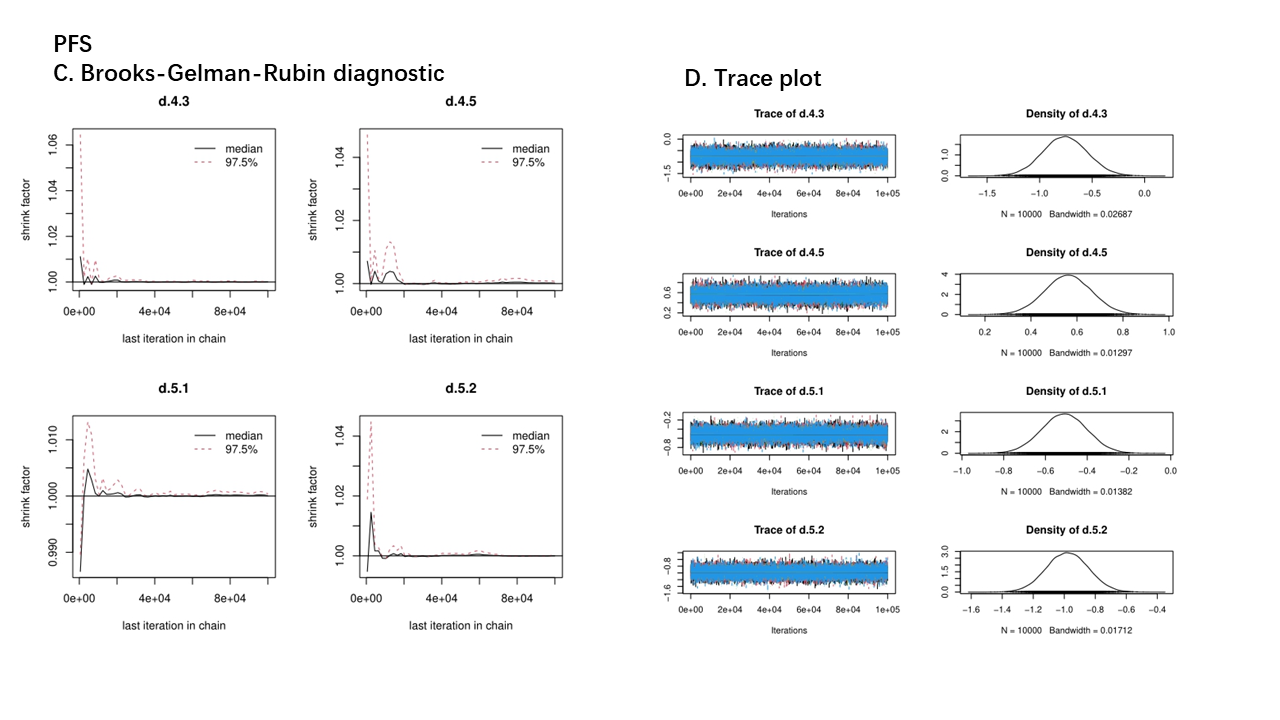

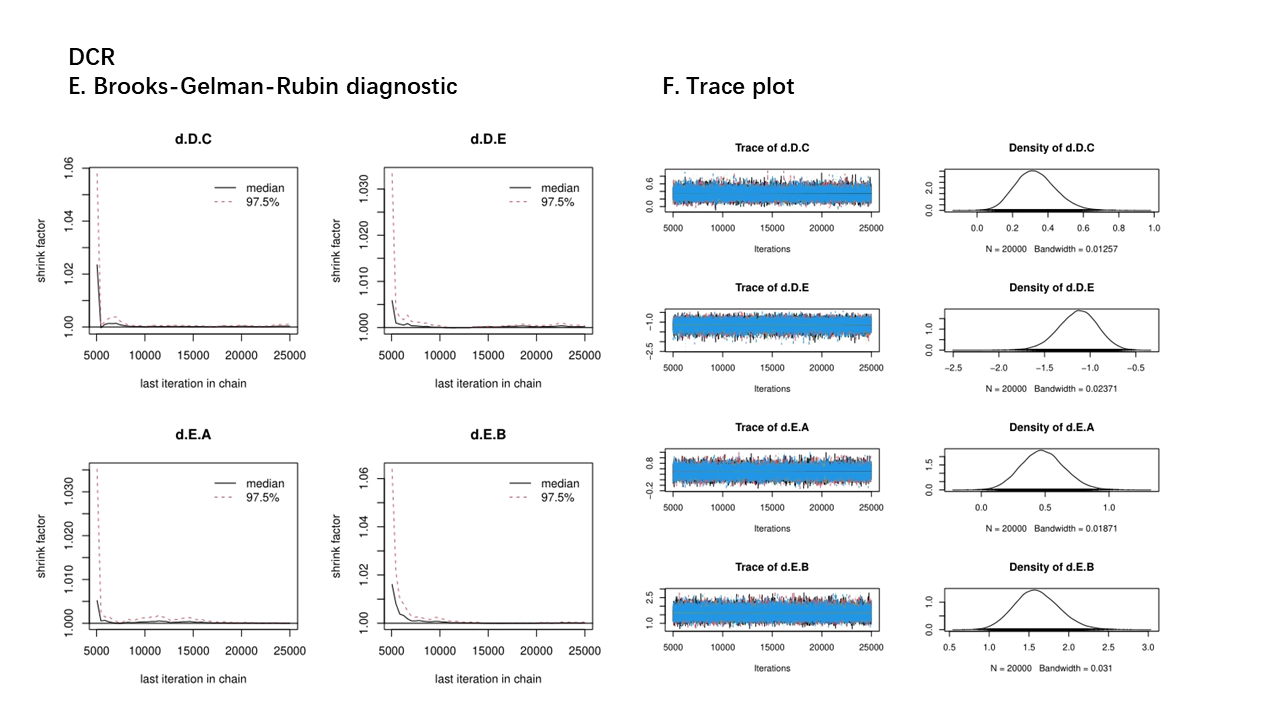

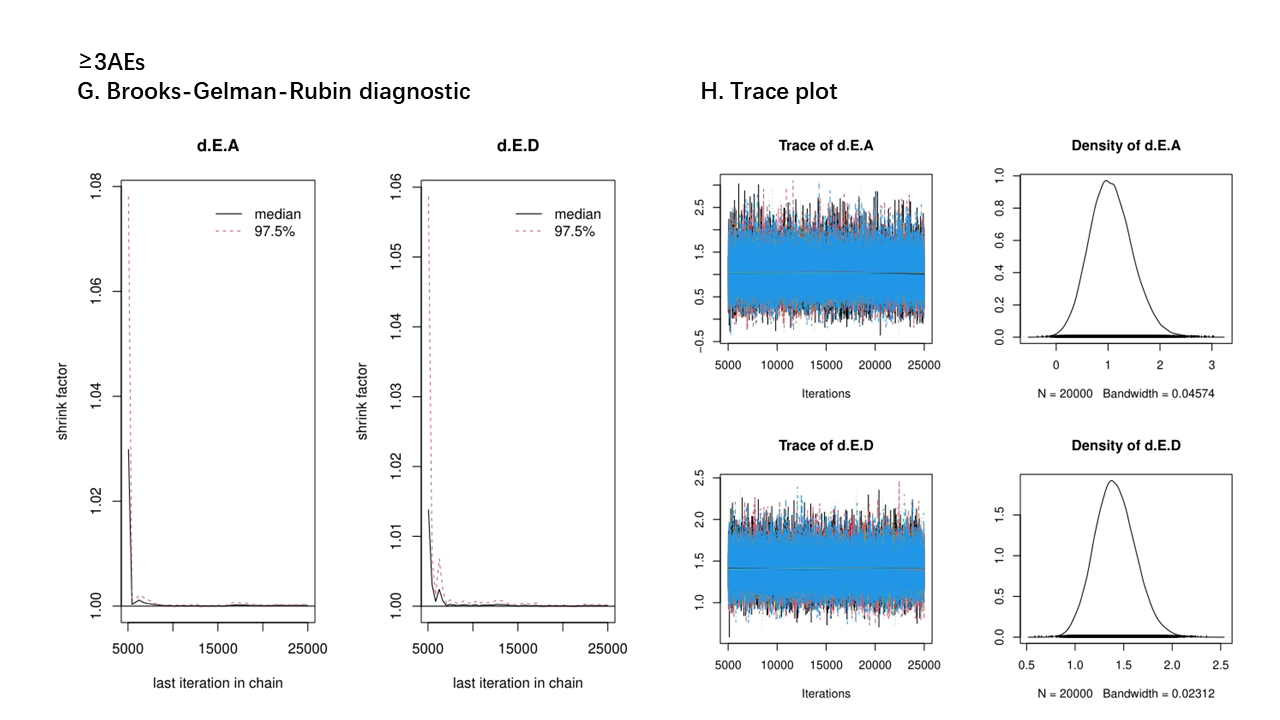

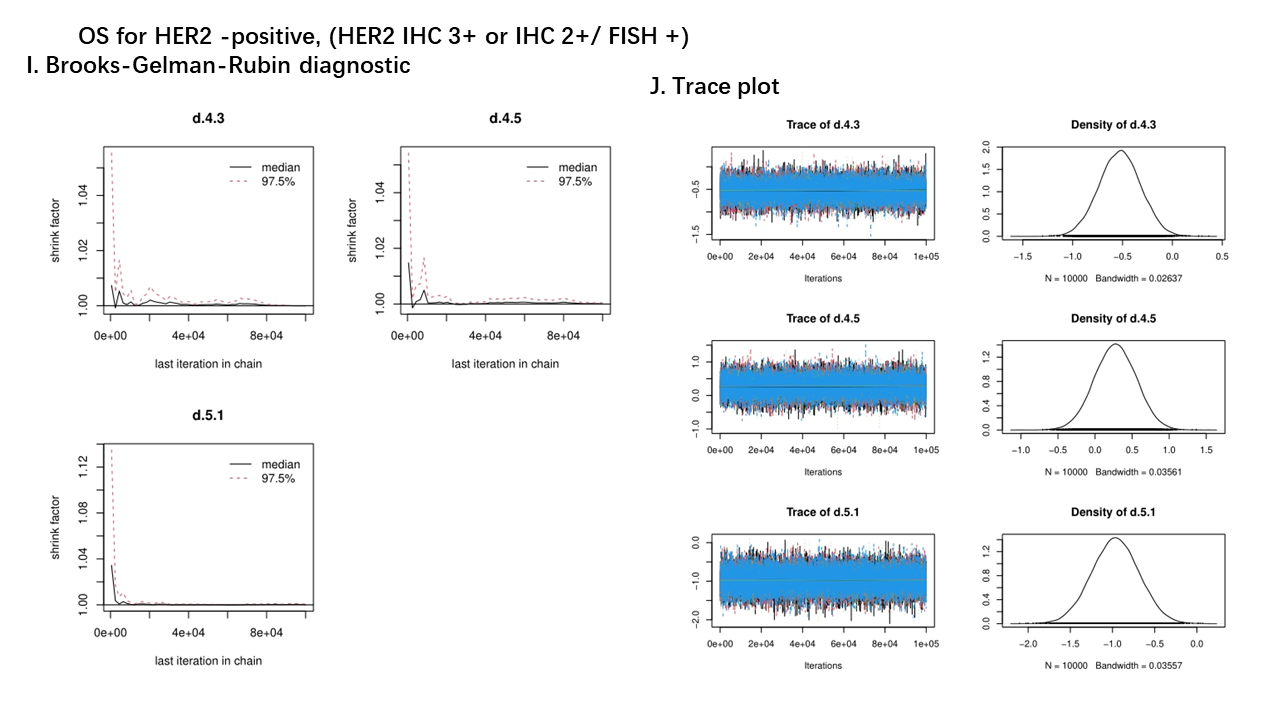

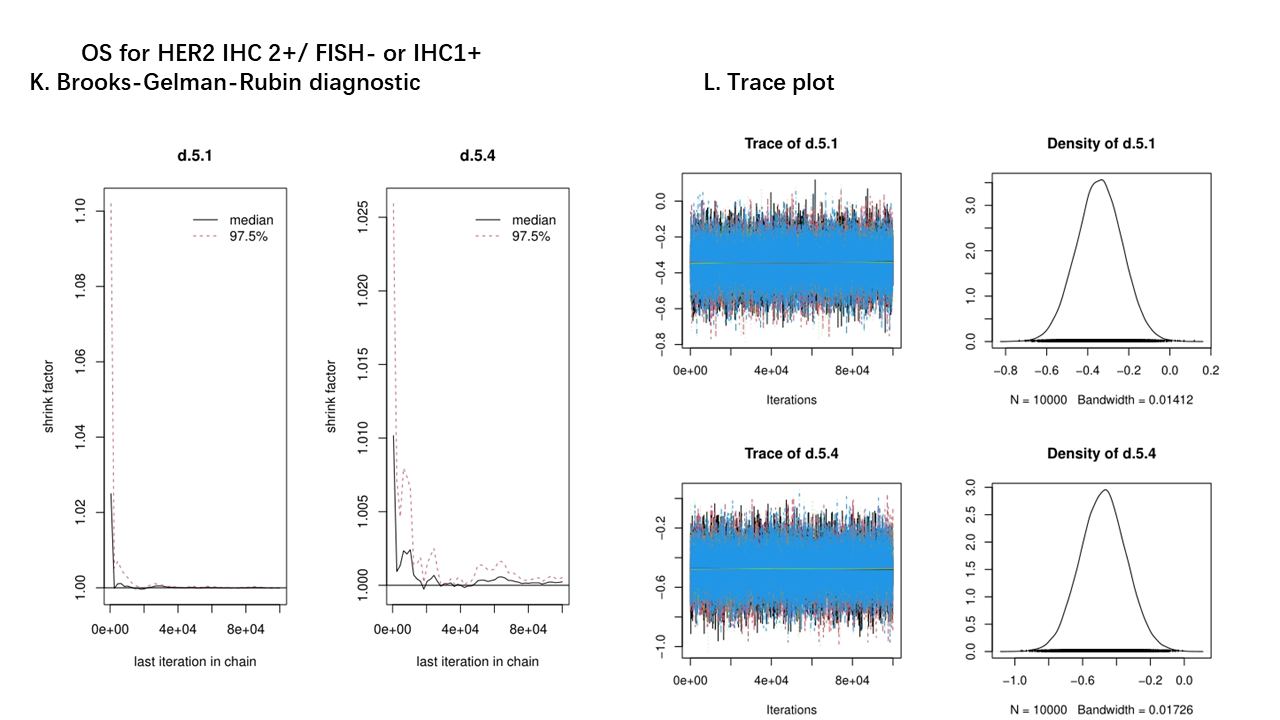

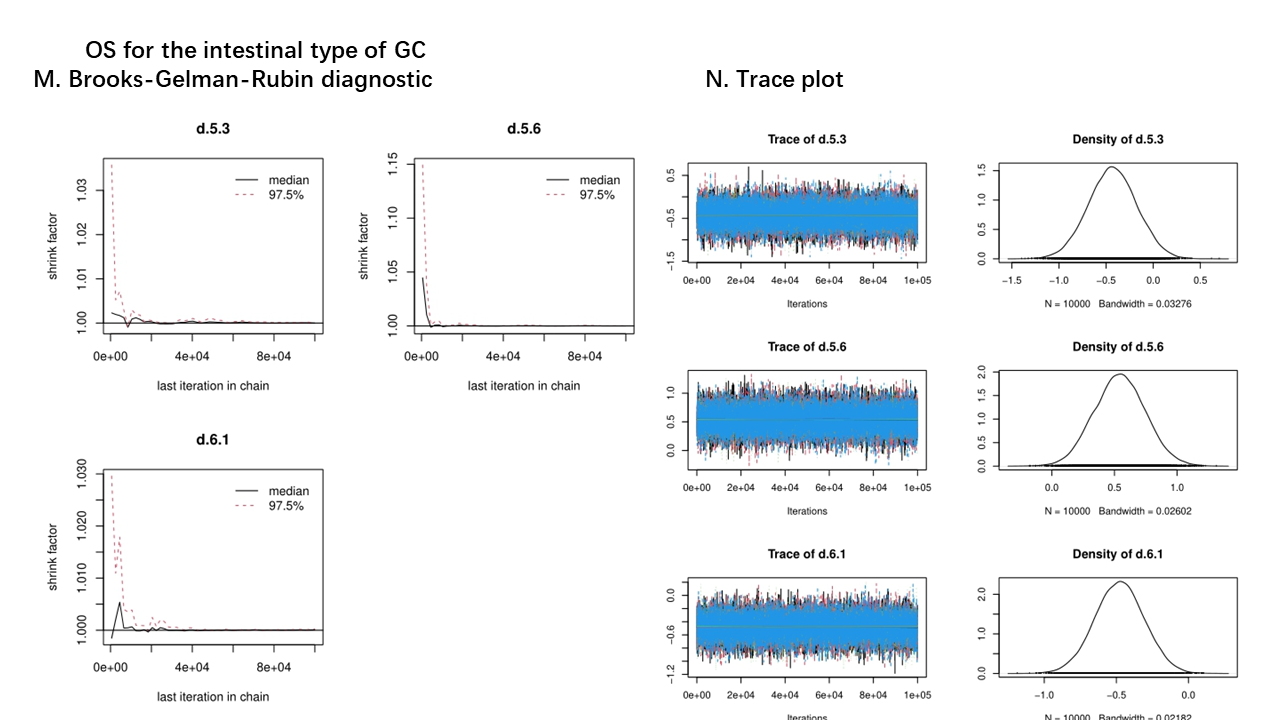

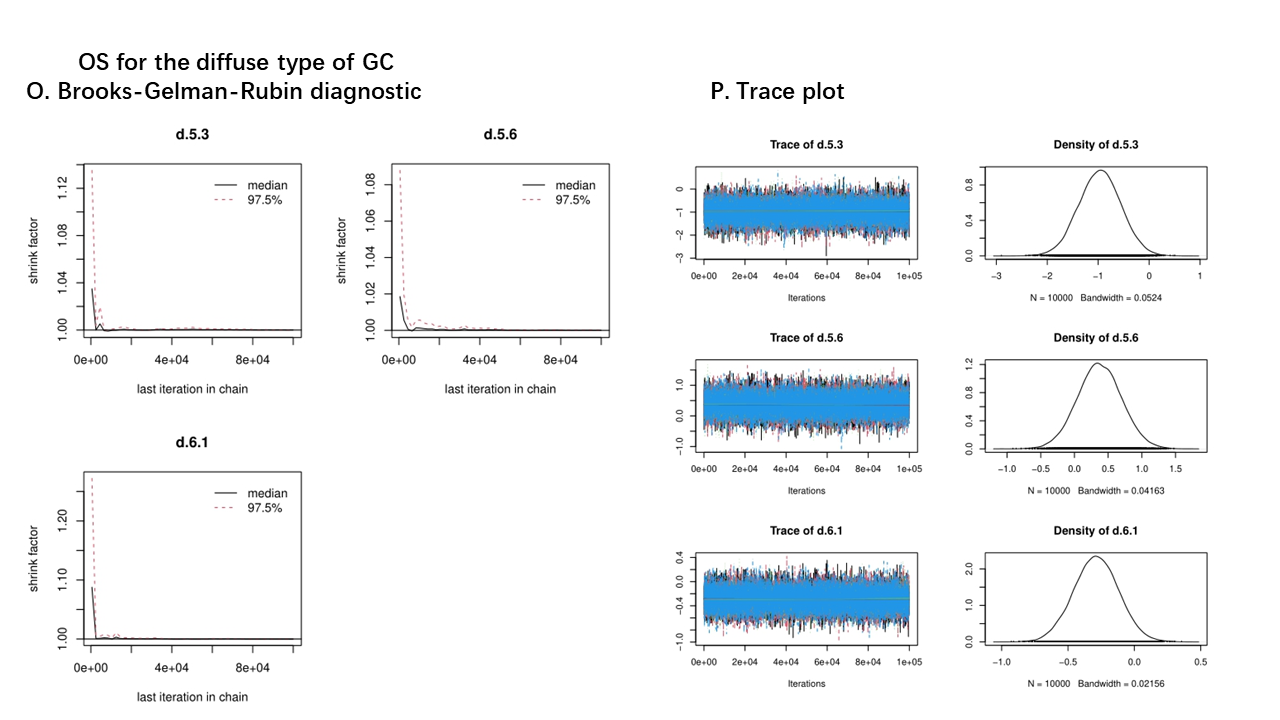


**Supplementary Figure 7**

Convergence of the four chains established by inspection of the Brooks-Gelman-Rubin diagnostic and the density trace plot. Overall survival (A and B), Progression-free survival (C and D), disease control rate (E and F), Adverse events of grade 3 or higher (G and H), HER2 -positive, (HER2 IHC 3+ or IHC 2+/ FISH +) (I and J), HER2 IHC 2+/ FISH- or IHC1+ (K and L), intestinal type of GC (M and N), the diffuse type of GC (O and P)


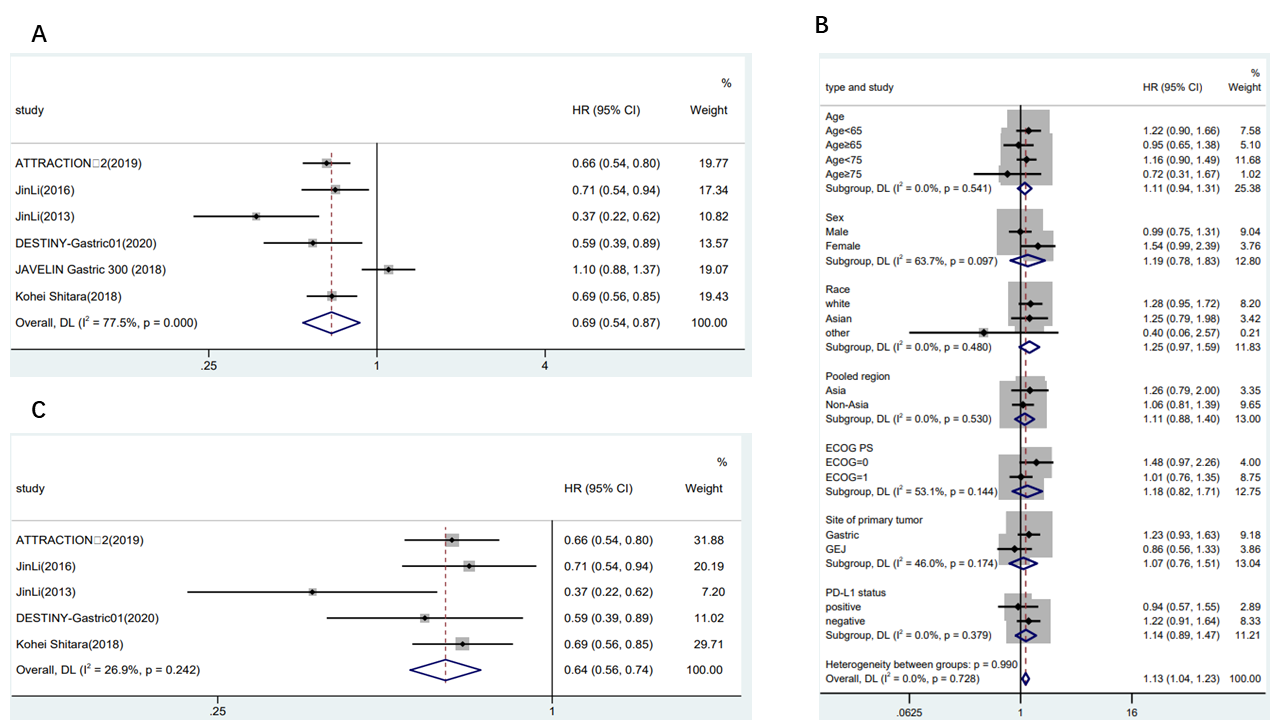


**Supplementary Figure 8**


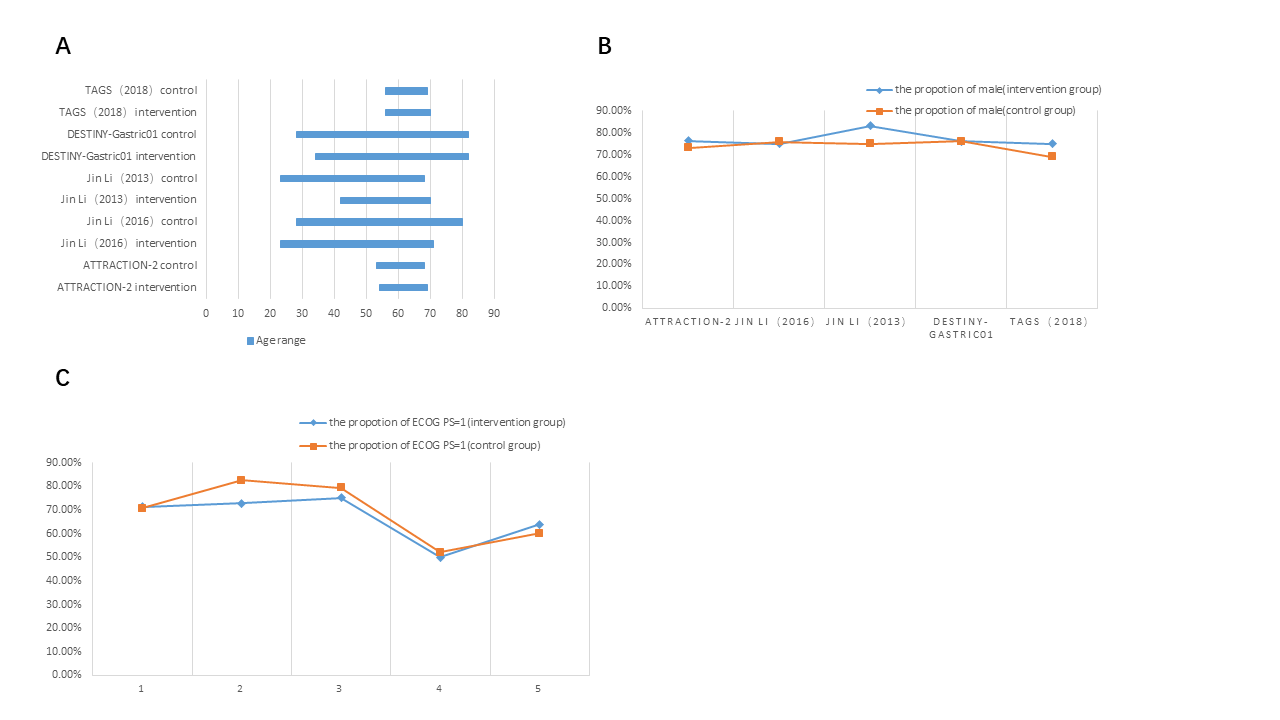
The heterogeneity test between the available RCTs(A) and finally included RCTs(C), Subgroup Analysis of Heterogeneity Test for JAVELIN Gastric300(2018) (B)Heterogeneity was assessed between studies using the I^2^ statistic. The estimated I^2^ values under 25%, between 25% and 50%, or over 50% indicated low, moderate, or high heterogeneity, respectively

**Supplementary Figure 9**

Assessment of transitivity. (A) Median age and range of patients in intervention and control groups. (B)

The proportion of male patients in intervention group and control group. (C)The proportion of ECOG PS=1 patients in intervention group and control group. ECOG PS, Eastern Cooperative Oncology Group Performance Status.
